# Efficacy and acceptability of interventions for co-occurring PTSD and SUD: A meta-analysis

**DOI:** 10.1016/j.janxdis.2021.102490

**Published:** 2021-10-26

**Authors:** Tracy L. Simpson, Simon B. Goldberg, Diana K.N. Louden, Shannon M. Blakey, Sage E. Hawn, Aline Lott, Kendall C. Browne, Keren Lehavot, Debra Kaysen

**Affiliations:** a VA Puget Sound Center of Excellence in Substance Addiction Treatment and Education, 1660 South Columbian Way, Seattle, WA 98108, USA; b University of Washington, Department of Psychiatry, 1959 NE Pacific St, Seattle, WA 98195, USA; c Department of Counseling Psychology, University of Wisconsin - Madison, 1000 Bascom Mall, Madison, WI 53706, USA; d University of Washington, Health Sciences Libraries, 1959 NE Pacific St, Seattle, WA 98195, USA; e Durham VA Medical Center, 508 Fulton St, Durham, NC 27705, USA; f VA Mid-Atlantic Mental Illness Research, Education and Clinical Center, 3022 Croasdaile Dr, Durham, NC 27705, USA; g Boston VA Medical Center, 150S Huntington Ave, Boston, MA 02130, USA; h VA Puget Sound Seattle/Denver HSR&D COIN, 1660 South Columbian Way, Seattle, WA 98108, USA; i Stanford University, Department of Psychiatry, 401 Quarry Road, Stanford, CA 94305, USA

**Keywords:** Meta analysis, Posttraumatic stress disorder, Substance use disorder, Randomized controlled trials, Cognitive behavior therapy

## Abstract

Over the past 20 years, numerous treatments addressing comorbid Posttraumatic Stress Disorder (PTSD) and Substance Use Disorder (SUD) have been developed and tested. The current meta-analysis examined the efficacy and acceptability of the two central treatment types– trauma-focused and non-trauma-focused – compared with all comparators and with cognitive-behavioral manualized SUD treatments immediately post-treatment and at longest follow-up. Twenty-eight randomized clinical trials (*N* = 3247) were included. There were small to large within-group effects for all forms of active treatment (*g*s = 0.30–1.11). Trauma-focused but not non-trauma-focused treatments outperformed all comparators on PTSD outcomes at post-treatment. Neither trauma-focused nor non-trauma-focused treatment outperformed all comparators on SUD outcomes at post-treatment. Neither trauma- nor non-trauma-focused treatment outperformed manualized SUD treatments on PTSD outcomes at either time point. Manualized SUD treatments outperformed trauma-focused treatments on SUD outcomes at post-treatment and non-trauma-focused treatments on PTSD outcomes at follow-up. Regarding treatment retention, neither trauma-focused nor non-trauma-focused treatments significantly differed from all comparators or from manualized SUD treatments. Between-group results were largely unchanged in trim-and-fill analyses, but were not robust to fail-safe N. Few moderators were detected. Taken together, results suggest that trauma-focused, non-trauma-focused, and manualized SUD interventions are sound options for individuals with comorbid PTSD/SUD.

## 1. Introduction

Posttraumatic Stress Disorder (PTSD) is the hallmark psychiatric disorder that can develop following trauma exposure (American Psychiatric Association, 2013). Past-year and lifetime prevalence estimates of PTSD among community-dwelling U.S. adults are 4.7% and 6.1%, respectively (Goldstein et al., 2016). PTSD commonly co-occurs with other psychiatric conditions, including Substance Use Disorders (SUDs). Recent epidemiologic research demonstrates that 57.7% of those with lifetime PTSD have had either a lifetime Alcohol Use Disorder (AUD), Drug Use Disorder (DUD), or both, while the burden of lifetime PTSD among those with lifetime SUD is markedly lower (12.3%; Simpson, Rise, Browne, Lehavot, & Kaysen, 2019; see also Blanco et al., 2013). Among those seeking SUD treatment, PTSD comorbidity is, however, quite common, with estimates of current PTSD generally being just under 40% (Gielen, Havermans, Tekelenburg, & Jansen, 2012; Harrington & Newman, 2007; Reynolds et al., 2005). Critically, individuals with co-occurring PTSD and SUD are at elevated risk relative to their single disorder peers regarding social and functional instability, additional psychiatric comorbidities, and risk of suicidal behaviors (Blanco et al., 2013; Hassan, Le Foll, Imtiaz, & Rehm, 2017; Sells et al., 2016).

Given the level of disease burden and deleterious effects on functioning associated with co-occurring PTSD/SUD, it is not surprising that these individuals are likely to access treatment. A recent nationally representative study estimated that approximately 36% of people with comorbid PTSD/SUD have sought SUD-specific care while 84% have sought care in mental health settings (Simpson et al., 2020; see also Hawn, Cusack, & Amstadter, 2020). However, despite these high rates of treatment engagement, persistence of PTSD is common with over two-thirds of individuals with comorbid PTSD/SUD meeting criteria for a chronic PTSD diagnosis and nearly 40% for a chronic SUD (i.e., positive diagnosis both prior to the past year and in the past year; Simpson et al., 2020).

Such epidemiological data highlight the under- and unmet treatment needs of people with comorbid PTSD/SUD and suggest that many of these individuals do not adequately respond to available treatments at the population level. However, epidemiological data are not fine-grained enough to provide specific insights regarding the types of care associated with better and worse outcomes, as information regarding dose and type of care (e.g. whether empirically supported) are generally not available. Rather, we must rely on the results of randomized clinical trials (RCTs) and synthesis of these results. Although many RCTs examining PTSD interventions have excluded people with SUDs (Leeman et al., 2017), there is still a substantial body of relevant work pertaining to treatment options for individuals with PTSD/SUD.

Broadly, behavioral interventions that have been evaluated as treatments for PTSD/SUD in RCTs may be classified as either (a) *trauma-focused treatments*, meaning that systematic trauma-processing occurs in the majority of sessions (Concurrent Treatment of PTSD and Substance Use Disorders Using Prolonged Exposure; COPE; Back et al., 2018; Norman et al., 2019; Cognitive Processing Therapy [CPT]; Simpson et al., 2021; and Prolonged Exposure therapy [PE]; Foa et al., 2013) or (b) *non-trauma-focused treatments* that address both SUD and PTSD without explicit discussion of trauma specifics (Integrated Cognitive Behavioral Treatment [ICBT]: McGovern et al., 2015; Seeking Safety: Najavits, Krinsley, Waring, Gallagher, & Skidmore, 2018). All of the extant non-trauma-focused treatments address PTSD symptoms and substance use in various integrated fashions while approximately half of trauma-focused treatments integrate elements aimed at ameliorating SUDs with trauma-exposure elements to address PTSD symptomatology (Back et al., 2019; Ruglass et al., 2017) and half deliver the SUD and PTSD treatments separately (Coffey et al., 2016; Foa et al., 2013).

There have been robust efforts to synthesize the available literature on PTSD/SUD behavioral treatments. Indeed, there are numerous narrative reviews (Bailey, Trevillion, & Gilchrist, 2019; Berenz & Coffey, 2012; van Dam et al., 2012; Flanagan et al., 2016; McCauley, Killeen, Gros, Brady, & Back, 2012; Najavits & Hien, 2013; Ralevski, Olivera-Figueroa, & Petrakis, 2014; Simpson, Lehavot, & Petrakis, 2017) and two meta-analyses (Roberts, Roberts, Jones, & Bisson, 2015; Torchalla, Nosen, Rostam, & Allen, 2012) of this literature. Roberts et al. (2015) included 13 RCTs in the most recent and comprehensive meta-analysis. They found trauma-focused treatments to be significantly more effective than treatment-as-usual and no/minimal treatment for PTSD both at post-treatment and follow-up and for substance use at follow-up, but also associated with significantly poorer treatment retention, again compared to treatment-as-usual and no/minimal treatment. Non-trauma-focused treatments did not differ significantly from comparators on treatment retention and little support was found for them on either PTSD or SUD indicators.

While Roberts et al. (2015) meta-analysis provided a valuable overview of the extant PTSD/SUD behavioral RCT literature, the number of relevant studies has more than doubled in the intervening six years. Given the modest number of studies available for several comparisons tested by Roberts et al. (*k*s ranged from 1 to 4 studies), which likely impacted statistical power (Valentine, Pigott, & Rothstein, 2010), the increase in available studies suggests that it is an opportune time for an updated examination. In addition, Roberts et al. did not evaluate potential moderators of treatment response, such as participant drug use involvement or recruitment setting (i.e., SUD treatment clinics vs. community-based recruitment), factors that may be markers of disease severity (Moss, Goldstein, Chen, & Yi, 2015; Simpson et al., 2020). Thus, we undertook an updated meta-analysis of behavioral treatments for adults with co-occurring PTSD/SUD in RCTs involving outcomes pertaining to PTSD severity, substance use, and treatment retention. Following Roberts et al., we classified studies as testing trauma-focused treatments or non-trauma-focused treatments and examined outcomes at both immediate post-treatment and longest follow-up.

In addition to comparing trauma-focused treatments to all comparators and non-trauma-focused treatments to all comparators (manualized SUD treatment, SUD treatment as usual, and no/minimal treatment), we also included comparisons involving only those trials that included manualized SUD treatments. The latter set of comparisons was undertaken because manualized SUD treatment conditions generally account for time, attention, and therapist training, thus allowing evaluation of the unique contributions of trauma-focused and non-trauma-focused treatments above and beyond common therapeutic elements (Wampold & Imel, 2015). For trauma-focused interventions, unique therapy elements typically include explicit focus on trauma memories to provide an opportunity for new learning and emotional processing (PE; Foa et al., 2013) or to arrive at healthier beliefs about the trauma, oneself, and others (CPT; Resick, Monson, & Chard, 2016). For non-trauma-focused interventions, unique therapy elements typically include strategies to increase patients’ understanding of the relationships between trauma-related triggers and substance use and to build coping skills to enable patients to break this reactive link (ICBT, McGovern et al., 2015; Seeking Safety, Najavits et al., 2018). Also, because manualized SUD treatments are frequently offered in SUD clinical settings (Center for Substance Abuse Treatment, 1999; Hendershot, Witkiewitz, George & Marlatt, 2011; VA/DoD Management of Substance Use Disorders Workgroup, 2015), these interventions are likely received by many with PTSD/SUD who do not have access to care for both aspects of the comorbidity because few community-based clinics offer PTSD/SUD treatment (see Killeen, Back, & Brady, 2015). Although such interventions are likely delivered more rigorously in the context of RCTs than in standard clinical settings, knowledge of the efficacy of manualized SUD treatments for individuals with co-occurring PTSD/SUD is important from a public health standpoint. Specifically, should meta-analytic empirical research suggest that manualized SUD treatments are either comparable or superior to either trauma-focused or non-trauma-focused PTSD/SUD treatments it would support investing in improving the delivery of these already familiar care options.

We hypothesized that trauma-focused treatments would be associated with superior PTSD and substance outcomes relative to all comparators, but would outperform manualized SUD treatment only on PTSD outcomes. Based on Roberts et al. (2015) findings, we anticipated that trauma-focused treatments would be associated with significantly lower treatment retention than all comparators generally and manualized SUD treatments specifically. We did not anticipate differences between non-trauma-focused treatments and all comparators or manualized SUD comparators on PTSD or SUD outcomes or treatment retention (Roberts et al., 2015). In the absence of data explicitly speaking to treatment acceptability, we followed Roberts et al. (2015) and used treatment retention as a proxy for treatment acceptability.

We examined several potential moderators. First, because there is consistent support for the self-medication hypothesis in the context of co-occurring PTSD/SUD such that many individuals with both disorders report using substances to cope with their PTSD memories and symptoms (Hawn et al., 2020) and those with comorbid PTSD/SUD generally prefer integrated treatment (Back et al., 2014), we evaluated whether integrated trauma-focused treatment that addresses both sides of the comorbidity simultaneously (vs. non-integrated) moderated treatment outcomes.^1^ Second, in light of findings suggesting that those with co-occurring PTSD/DUD are at elevated risk of worse social functioning (e.g., greater risk of unemployment and lifetime incarceration; lower educational attainment), greater SUD severity, and greater suicide risk than those with PTSD/AUD-only (Simpson et al., 2019), we evaluated whether the proportion of the samples with reported drug use moderated treatment effects. Third, we explored whether the setting in which the RCTs took place (e.g., SUD treatment clinics vs. community recruitment by university-based investigators) moderated outcomes given epidemiologic evidence that those with PTSD/SUD who receive SUD treatment have markedly more severe SUDs and are more likely to have chronic SUDs than those reporting no treatment or mental health treatment only (Simpson et al., 2020). Fourth, we evaluated whether differential assessment attrition moderated outcomes because study outcomes could be biased if, for example, less severe, more stable study participants are more likely to attend post-treatment assessments than more severe, less stable participants. Finally, we examined whether treatment delivery platform moderated outcomes because individually delivered treatment has been found to be more efficacious in treating PTSD than group delivered treatment (Haagen, Smid, Knipscheer, & Kleber, 2015; Resick et al., 2017).

## 2. Method

### 2.1. Protocol and registration

No published protocol exists for this meta-analysis.

### 2.2. Eligibility criteria

Studies needed to meet the following basic criteria to be included: (1) were available in English, (2) examined the effects of cognitive and/or behavioral treatments (i.e., psychotherapies) with the intention of addressing both PTSD and SUD, (3) included only adults ages 18 and over (4) used an RCT design, and (5) outcomes pertained to both PTSD symptomatology and substance use. Study samples also needed to be comprised of individuals with current comorbid PTSD and SUD such that at least 80% of participants met diagnostic criteria for both disorders or at least 80% had clinical presentations consistent with PTSD and SUD. Specifically, studies involving a mix of participants with threshold and sub-threshold DSM or ICD diagnoses of PTSD were included as were studies with participants who screened positive for PTSD (i.e., if diagnostic interviews were not conducted). Of note, ample evidence suggests that subthreshold PTSD is associated with significant distress and impairment (Pietrzak, Goldstein, Southwick, & Grant, 2011; Zlotnick, Franklin, & Zimmerman, 2002). Similarly, studies were included if they used an accepted alcohol or drug use screen (e.g. the AUDIT; Babor, de la Fuente, Saunders, & Grant, 1992) with a cut score indicating likely disordered use AND required recent unsafe use (e.g. alcohol consumption in excess of NIAAA safe drinking guidelines).

No restrictions were placed on publication status (i.e., unpublished data and dissertations were eligible). Studies were excluded if they only assessed ancillary indicators of substance use (e.g. craving), did not assess both outcomes in an outpatient context (e.g. all assessments took place in controlled environments), or reported on subsets of samples from studies not intending to address both aspects of the comorbidity during treatment (e.g., Jason et al., 2011; Park, Cheng, Samet, Winter & Saitz, 2015).^2^ We contacted investigators regarding the availability of results from unpublished RCTs identified in trial registries (e.g., Hamblen, personal communication; Vujanovic, personal communication) as well as for information on subsets of participants likely meeting criteria for PTSD/SUD in studies targeting these disorders but allowing a broader spectrum of enrollees (i.e., Brief et al., 2013, personal communication; Haller et al., 2016, personal communication).

### 2.3. Information sources

The Preferred Reporting Items for Systematic Reviews and Meta-Analyses (PRISMA) standards were followed (Moher, Liberati, Tetzlaff, Altman & PRISMA Group, 2009; see Table A.1 for PRISMA Checklist). With input from the first author (TLS), a medical research librarian (DKNL) systematically searched ten databases: PubMed, Embase, Cochrane Central Register of Controlled Trials, PsycINFO, CINAHL Complete, PTSDpubs, Web of Science, ProQuest Dissertations and Theses Global, ClinicalTrials.gov, and the International Clinical Trials Registry Platform. Databases were searched from inception to July 9, 2021. Five articles were identified for review through review of reference sections.

### 2.4. Search, study selection, and data collection process

The search strategies incorporated controlled vocabulary terms and keywords appropriate to each database to represent the concepts of PTSD, SUD, and controlled clinical trials (see Table A.2 for full search terms used in each database).

After removing duplicates, each title and/or abstract was independently evaluated by two of three authors (SEH, AL, TLS) based on inclusion/exclusion criteria. The remaining studies underwent full text review by two of the same three authors. Disagreements were settled through discussion until consensus was reached.

Two of three authors independently extracted information on demographics, treatment delivery, and outcome data for each study using standardized spreadsheets (SEH, AL, TLS). There were no substantive errors detected in the coding when the spreadsheets were mechanically compared. Two authors (SMB, TLS) independently extracted risk of bias information with disagreements discussed until consensus was reached.

### 2.5. Data items

Data necessary for computing effect sizes from continuous measures (e.g. number of drinking days) included sample size, means, and standard deviations. For dichotomous outcomes (e.g. number of participants reporting drug use), we extracted sample sizes in the relevant cells. Information on all relevant outcomes was recorded. For example, if a study reported both average drinks per drinking day and percent days heavy drinking, both measures were included. Symptom outcome assessment data were recorded for baseline, immediate post-treatment, and the longest follow-up. We restricted the meta-analytic data to these two post-treatment assessments so that the level of precision across studies was similar.

In addition, we extracted demographic and clinical features of the samples and characteristics of the treatment and control conditions. These included mean age, percentage female, percentage racial/ethnic minority, percentage meeting full PTSD diagnostic criteria, percentage meeting SUD diagnostic criteria, percentage with drug use, treatment and control condition name and type, intention-to-treat (ITT) sample size, treatment length (number of sessions or weeks), number of weeks and/or sessions required for treatment completion, percentage of participants completing treatment, treatment modality (e.g., group, individual), and treatment setting (e.g., SUD treatment clinic, residential SUD treatment, university laboratory).

Conditions were categorized into the following five groups: (1) trauma-focused, (2) non-trauma-focused, (3) CBT manualized SUD (4) SUD TAU, or (5) no/minimal treatment. All of the identified studies were included in preliminary within-subject models evaluating changes in outcomes over time associated with these five categories. Between-group comparisons that involved either trauma-focused or non-trauma-focused treatments compared with any of the other three categories (i.e., manualized SUD, SUD TAU, no/minimal treatment) were included in the between-group models. A separate set of between-group models compared either trauma-focused or non-trauma-focused treatments with manualized SUD treatments only.^3^

We followed Fu et al. (2011) guidance that four or more studies are needed for meta-analysis and thus did not conduct between-group tests on the two studies that included comparisons between trauma-focused and non-trauma-focused treatments (Najavits et al., 2018; Norman et al., 2019) in separate models. These studies were excluded from the primary between-group analyses but were included in sensitivity analyses in the trauma-focused vs. all comparators models. To isolate the effects of trauma-focused treatments involving empirically supported PTSD treatment components (CPT: Simpson et al., 2020; Vujanovic personal communication; PE^4^: Back et al., 2019; Foa et al., 2014; Ruglass et al., 2017) relative to the most rigorous comparators (manualized SUD), a set of sensitivity analyses involving only those studies were conducted.

### 2.6. Risk of bias in individual studies

Individual studies were rated with the Cochrane risk-of-bias tool (Higgins & Green, 2008). Bias was assessed across six domains including: selection bias (random sequence generation, allocation concealment), detection bias (masking of outcome assessors), attrition bias (use of intent-to-treat models that account for missing data), reporting bias (selective reporting), and other sources of bias (baseline imbalance, incomplete reporting of methods). Risk of bias was assessed as low, high, or unclear on each domain for each study.

### 2.7. Summary measures

Standardized effect sizes were calculated using standard meta-analytic methods (Borenstein, Hedges, Higgins, & Rothstein, 2009). For continuous outcomes, this involved first computing a within-group pre-post (or pre-follow-up) Cohen (1988). We assumed a correlation of *r*_xx_ = 0.50 between time points (which is lower than a typical test-retest correlation to account for potential changes due to intervention; Hoyt & Del Re, 2018). Between-group effects were calculated as the difference between the within-group effects (i.e., Becker, 1988] δ). This method has the advantage of incorporating baseline data, as opposed to using post-test only. For categorical outcomes, we computed odds ratios (OR) reflecting the likelihood of a given outcome (e.g. drug use) based on group status (i.e., treatment or comparator). ORs were converted into Cohen’s *d*-units using standard methods (Borenstein et al., 2009) and incorporated into the between-group effect estimates. Effect sizes were converted into Hedges’ *g* to account for small sample bias (Cooper, Hedges, & Valentine, 2009). The sign for each effect was adjusted so that a positive effect size always indicated improvement and/or larger improvement in the treatment group relative to the control condition (i. e., decreased symptoms). We calculated treatment retention for studies that reported these data for both the treatment and control conditions. We computed an OR reflecting the likelihood of completing treatment for those assigned to the treatment conditions relative to those assigned to the control conditions.

### 2.8. Synthesis of Results

Using standard meta-analytic methods (Cooper et al., 2009), effects were next aggregated within measure (e.g. across subscales of the Addiction Severity Index) and then within study using the ‘agg’ function from the ‘MAd’ package (Del Re & Hoyt, 2014) in R (R Core Team, 2018). Separate omnibus analyses were conducted to characterize within-group and between-group change at post-treatment and follow-up on PTSD or SUD symptoms and for between-group differences on treatment retention. Effects on symptoms were examined across the five within-group categories and four between-group comparisons. Thus, 36 aggregate effect sizes are reported (i.e., 2 ×2 X 9). An additional four effect sizes assessed differential treatment attrition in the four between-group categories.

Some studies included multiple treatment or control groups (e.g. Foa et al., 2013). For the within-group models, each treatment and control arm contributed a unique effect size. For the between-group models we balanced using all available data without duplicating data because duplicating data creates a unit-of-analysis error and violates assumptions of non-independence (Higgins & Green, 2008). For the study that included four arms (two treatment, two control; Foa et al., 2013), we computed separate between-group effects for two separate treatment and control pairings. For the study that included two trauma-focused treatment groups of the same type with and without motivational enhancement therapy and one control group (Coffey et al., 2016), we combined the two treatment groups into a single group. For the study with two active conditions and a time/attention placebo (Stappenbeck et al., 2015), we included the CBT intervention (i.e., Cognitive Restructuring) rather than the mindfulness-oriented intervention. For the study with two active conditions (Simpson et al., 2021; CPT and Relapse Prevention) and a six-week assessment only condition prior to re-randomization to one of the active conditions, we included only the active conditions following re-randomization (and treated Relapse Prevention as a manualized SUD treatment).

Heterogeneity was characterized using I^2^ (i.e., proportion of variance that occurs between studies). Random effects models were conducted with weighting based on the inverse variance of each study’s effect size using the ‘metafor’ package (Viechtbauer, 2010).

### 2.9. Risk of bias across studies

Trim-and-fill analyses using the ‘metafor’ package assessed publication bias. When funnel plot asymmetry was detected, a trim-and-fill-adjusted effect size was calculated with studies imputed to account for asymmetry. We also calculated the fail-safe N (FSN; Rosenthal, 1979). This value is intended to represent the number of unpublished non-significant results that would need to exist to nullify an observed effect (i.e., the “file drawer problem”). Based on Rosenberg (2005) guidelines, effects were considered robust to publication bias based on FSNs that were greater than five times the number of available studies plus 10.

### 2.10. Additional analyses

We tested five potential moderators of treatment effects: assessment attrition, treatment delivery platform (group vs. individual), percentage of the sample that reported drug use (as defined by each study), study recruitment site (SUD treatment clinical setting vs. community-based recruiting), and whether a trauma-focused treatment was integrated with SUD content (for trauma-focused models only).

We conducted four sensitivity analyses. First, we re-ran all within-and between-group models omitting the one study that did not formally diagnose SUD (i.e., Brief et al., personal communication). Second, we evaluated whether adding Najavits et al. (2018) and Norman et al. (2019) to the trauma-focused vs. all comparators models changed the pattern of results. Third, we examined whether restricting the trauma-focused vs. manualized SUD treatment models to those that used empirically supported PTSD treatments (Back et al., 2019; Foa et al., 2013; Ruglass et al., 2018; Simpson et al., 2021; Vujanovic, personal communication) influenced the pattern of results. Finally, we also evaluated the impact of potential outliers. While there are several methods for identifying outliers in meta-analysis (Viechtbauer & Cheung, 2010), we implemented the ‘find.outliers’ function provided by Harrer, Cuijpers, Furukawa, and Ebert (2019). This function defines outliers as studies with confidence intervals that do not overlap the omnibus effect. Models were re-estimated with outliers excluded.

## 3. Results

### 3.1. Study selection

**Fig. 1.**
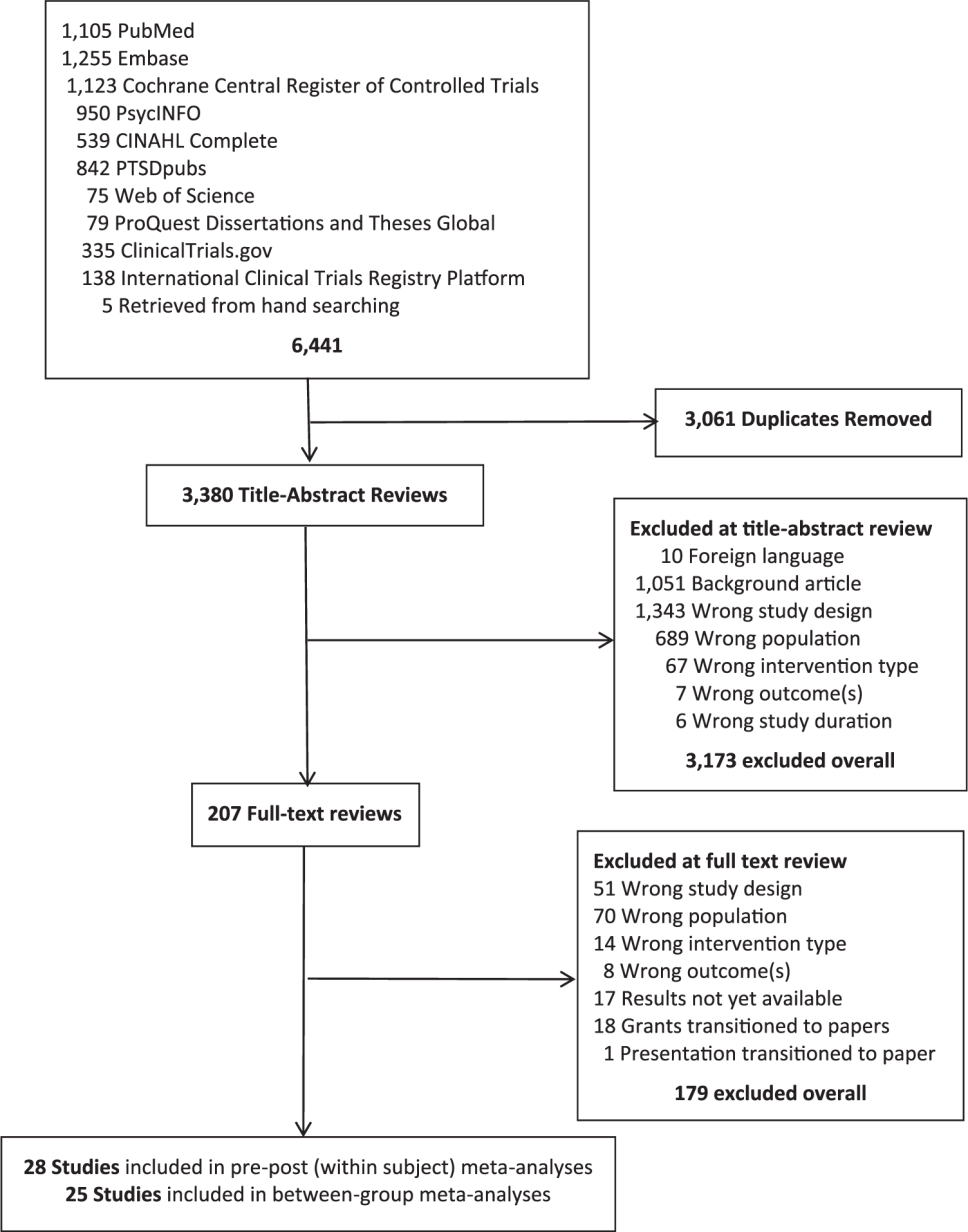
PRISMA flow diagram.

Our search yielded 6441 citations. After 3061 duplicates were removed, 3380 titles/abstracts were reviewed. We applied our inclusion/exclusion criteria, producing a final set of 28 studies (combined *n* = 3247; see Fig. 1). The earliest year of publication was 2004. Included studies are denoted with an asterisk in the reference section.

### 3.2. Study characteristics

Study-level characteristics are displayed in Table 1 and Table A.3 with summary statistics based on those with available data. There were 62 treatment and comparator arms across the studies that contributed to within-group effects with 19 trauma-focused, 16 non-trauma-focused, 12 manualized SUD treatment, 9 SUD TAU, and 6 no/minimal treatment. The names of all treatment and comparator conditions and their respective categories are listed in Table A.4. Overall, 24 comparisons contributed to between-group effects, 13 to trauma-focused vs. all comparators, and 11 to non-trauma-focused vs. all comparators. Among comparisons that controlled for time, attention, and treatment delivery rigor, 8 involved trauma-focused vs. manualized SUD treatments and 5 involved non-trauma-focused vs. manualized SUD treatments.

Sample sizes aggregated across all treatment and comparator conditions ranged from 12 to 386 (mean = 115.96, SD = 98.97). Treatment and comparator conditions lasted, on average, 11.69 weeks (SD = 4.34) and 15.64 sessions (SD = 7.44). On average, studies required attendance at 70.56% (SD = 22.73%) of sessions or weeks of treatment for participants to be considered completers. Collapsing across treatment completion indicators, overall, 55.01% (SD = 17.17) completed their assigned interventions, with an average treatment completion rate of 52.11% (SD = 20.86) for the trauma-focused treatments, 50.73% (SD = 10.27) for the non-trauma-focused treatments, and 55.95% (SD = 16.14) for the manualized SUD treatments (treatment completion was not relevant for SUD TAU and no/minimal treatment). The primary treatment modality was individual (81.82% of all treatments; 93.75% for trauma-focused, 73.33% for non-trauma-focused, and 100% for manualized SUD).

Participants’ average age was 40.21 (SD = 5.47), 38.88% were racial/ethnic minorities (SD = 22.92), and 46.90% were female (SD = 35.21). Most participants met full diagnostic criteria for PTSD (92.97%, SD = 11.93) and SUD (99.75%, SD = 1.01).^5^ Over half of participants reported current drug use (59.36%, SD = 24.97), although only 18 studies included this information.

### 3.3. Risk of bias within studies

Risk of bias varied across the studies, with 13 (53.57%) evidencing relatively low risk of bias as indicated by Cochrane bias scores of 5 or 6 out of 6 possible points (see Figure A.1 and Table A.5). Eleven (39.29%) studies scored 3 or less on the indicator. The domain at highest risk for bias was allocation concealment. Risk for bias was generally low for attrition bias (i.e., use of intent-to-treat analyses) and selective reporting. Whether randomization was carried out via random sequence generation was most frequently rated “unclear.”

### 3.4. Results of individual studies

Study-level effect size data are reported in Table A.6, separated by comparison, time point, and outcome domain. The specific outcome measures that were used to assess PTSD and SUD symptoms are listed by study in Table A.7.

### 3.5. Synthesis of results

**Fig. 2.**
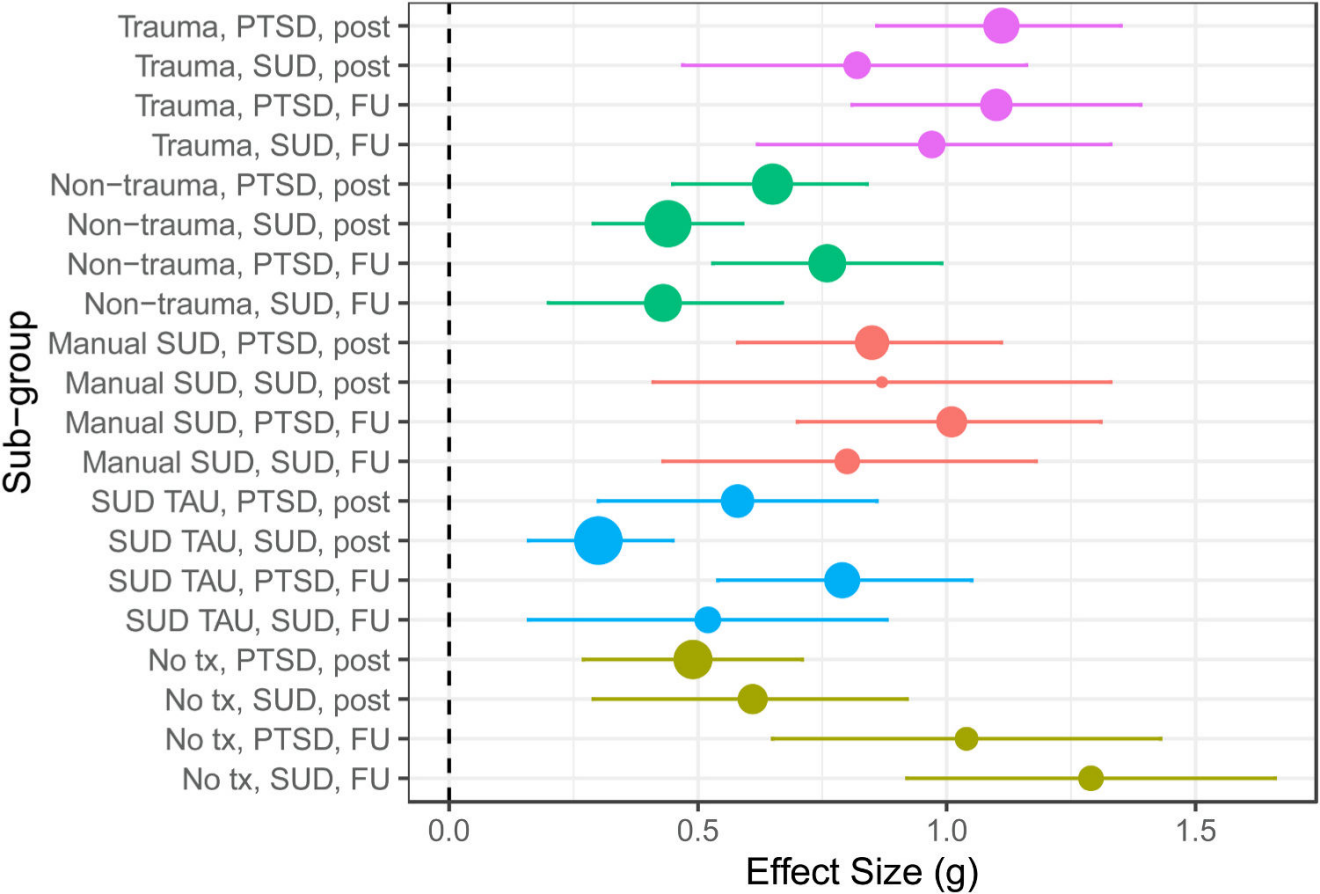
Forest plot of within-group effects separated by treatment type, domain, and time point. SUD = substance use disorder symptoms; Trauma = trauma-focused treatment; Non-trauma = non-trauma-focused treatment; Manual SUD = manualized SUD treatment; SUD TAU = SUD treatment-as-usual; No tx = no treatment control; FU = follow-up.

Within-group effects across the five treatment and control conditions are displayed in Table 2 and Fig. 2. All five types of conditions were associated with statistically significant small to large magnitude reductions in PTSD and SUD symptoms, both at post-treatment and follow-up. Heterogeneity was high across almost all within-group models (I^2^ > 75%).

**Fig. 3.**
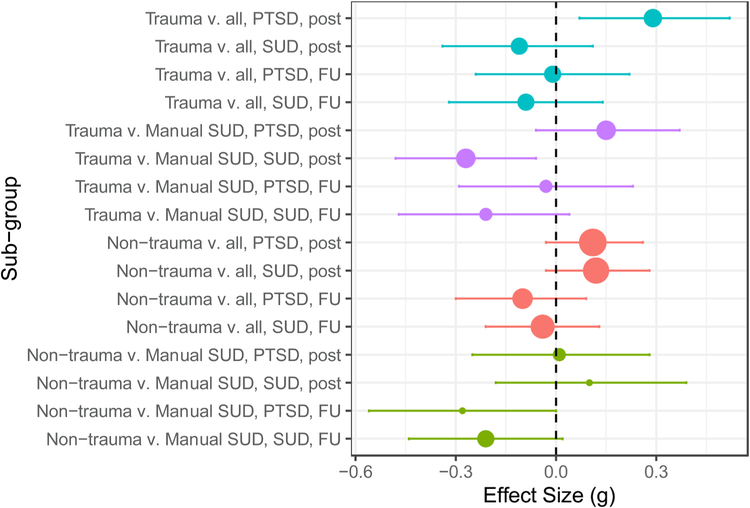
Forest plot of between-group effects separated by comparison, domain, and time point. Trauma = trauma-focused treatment; v. = versus; all = all comparators with the exception of trauma-focused or integrated non-trauma-focused; Manual SUD = manualized SUD treatment; Non-trauma = non-trauma-focused treatment; FU = follow-up. SUD = substance use disorder symptoms.

Between-group effects on PTSD and SUD outcomes as well as treatment completion are displayed in Tables 3 and 4 and Fig. 3. In general, these models indicated small magnitude differences that were not statistically significant between both trauma-focused and non-trauma-focused treatments when each was compared with all comparators and with manualized SUD treatments, with some exceptions described below.

Trauma-focused treatments showed significantly larger effects on PTSD at post-treatment (*g* = 0.29, [0.07, 0.52]) than all comparators, but this difference did not carry through to follow-up. Trauma-focused treatments did not differ from all comparison types on measures of SUD at post-treatment or follow-up. When trauma-focused treatments were compared with manualized SUD treatments there were no significant differences on PTSD at post-treatment or follow-up. Trauma-focused treatments showed significantly smaller effects on SUD symptoms than manualized SUD treatments at post-treatment (*g* = −0.27, [−0.48, −0.06]), but this difference did not persist to follow-up (*g* = −0.21, [−0.47, 0.04]). Trauma-focused treatments did not differ significantly from all comparators (OR = 0.85, [0.60, 1.21]) or manualized SUD treatments (OR = 0.92, [0.63, 1.36]; Table 4) on treatment completion. Sensitivity analyses revealed that none of the patterns in the between-group models changed when the two studies comparing trauma-focused and non-trauma-focused treatments (Najavits et al., 2018; Norman et al., 2019) were included. Similarly, the between-group patterns pertaining to trauma-focused vs. manualized SUD treatments were unchanged when only the five studies involving empirically supported PTSD treatments were considered (Back et al., 2019; Foa et al., 2013; Ruglass et al., 2017; Simpson et al., 2021; Vujanovic, personal communication).^6^

Heterogeneity varied somewhat across the between-group models for trauma-focused treatments and confidence intervals for I^2^ were generally large (Table 3). Heterogeneity was highest for comparisons with all treatments on PTSD outcomes at post-test (I^2^ = 32.95%). Heterogeneity was low (<25%; Higgins, Thompson, Deeks, & Altman, 2003) for several comparisons (e.g., with all comparators on PTSD at follow-up and SUD at post, with manualized SUD on PTSD and SUD at post and follow-up). Heterogeneity was low for treatment completion models (I^2^ = 0.00).

Non-trauma-focused treatments did not differ from all comparators on PTSD at post-treatment (*g* = 0.11, [−0.03, 0.26]) or follow-up (*g* = −0.10, [−0.30, 0.09]). Non-trauma-focused treatments also did not differ from all comparators on SUD outcomes at post (*g* = 0.12, [−0.03, 0.28]) or follow-up (*g* = −0.04, [−0.21, 0.13]). When compared to manualized SUD treatments, non-trauma-focused treatments did not differ significantly on either PTSD or SUD at post-treatment or follow-up, with one exception. Non-trauma-focused treatments were inferior to manualized SUD treatments on PTSD at follow-up (*g* = −0.28, [−0.56, −0.00], *p* = .048). Treatment completion rates did not differ between non-trauma-focused treatments relative to all comparators (OR = 1.22, [0.93, 1.60]) or manualized SUD treatments (OR = 1.24, [0.87, 1.78]).

Heterogeneity varied somewhat across the between-group models for non-trauma-focused treatments. It was highest for non-trauma-focused treatment versus all comparators on SUD outcomes at follow-up (I^2^ = 33.30%) and was low (I^2^ = 0.00%) in several models (e.g., vs. manualized SUD on PTSD at post and follow-up and SUD at follow-up).

### 3.6. Risk of bias across studies

Funnel plot asymmetry was detected in multiple models. However, statistical significance tests did not differ for trim-and-fill adjusted models, with four exceptions. Trauma-focused treatments no longer differed from all comparators on PTSD at post-treatment (adjusted *g* = 0.22, [−0.05, 0.49]) and non-trauma-focused treatments were inferior to manualized SUD on SUD symptoms at follow-up (adjusted *g* = −0.27, [−0.48, −0.05]; Table 3). Treatment completion was higher for non-trauma-focused treatments relative to all comparators (OR = 1.30, [1.00, 1.68], *p* = .046) and manualized SUD treatments (OR = 1.41, [1.01, 1.95]; Table 4, see Figs. A.2 and A.3 for corresponding funnel plots). FSN estimates indicate that all statistically significant within-group models and none of the between-group models were robust to publication bias.

### 3.7. Additional analyses

Results of moderator tests examining attrition, treatment delivery modality, percentage of participants reporting drug use, and study recruitment site are reported in Table A.8. In almost all models, these factors did not moderate effects. However, three within-group models showed a positive association between attrition and effect size estimates (i.e., higher attrition was associated with larger effect sizes). Group delivery format was associated with smaller effects in one within-group model. Higher percentage of participants reporting drug use was associated with larger effect sizes. Recruitment from SUD sites was associated with larger effects in five within-group models and two between-group models.

Results comparing effects of integrated and non-integrated trauma-focused treatments are reported in Table A.9. Integrated trauma-focused treatments showed smaller effects in two within-group models and three between-group models. Upon further examination, we found that none of the studies using an integrated trauma-focused treatment recruited from SUD clinics while 6 of 7 of studies using non-integrated trauma-focused treatments did. Therefore, meta-regressions were run controlling for recruitment site (SUD clinic vs. community-based). As shown in Table A.9, all five previously significant meta-regression coefficients were no longer statistically significant.

Sensitivity analyses pertaining to model results with the sole study that included participants with subthreshold SUD are reported in Table A.10. Significance tests for both within- and between-group models were unchanged.

Sensitivity analyses pertaining to model results with outliers removed are reported in Table A.11. Although outliers were detected in several within-group models and one between-group model, statistical significance tests did not change when they were omitted.

## 4. Discussion

The present meta-analysis synthesized results of the extant behavioral RCT literature pertaining to the treatment of adults with co-occurring PTSD/SUD. All within-group models showed significant effects, mostly in the moderate to large range, for both PTSD and SUD outcomes at follow-up. This pattern of results indicates that across both experimental (i.e., trauma-focused and non-trauma-focused treatments) and control conditions, participants in these studies generally improved on both primary outcomes. Within-group models show that trauma-focused and manualized SUD treatments were mostly associated with large effect sizes for both PTSD and SUD outcomes (*g*s = 0.80–1.11), while SUD TAU and non-trauma-focused treatments showed small to large effects (*g*s = 0.30–0.79). No/minimal treatment conditions were associated with small to large effects (*g*s = 0.49–1.29), though the large effects were based on the sole study with follow-up data wherein nearly all control participants were in SUD treatment (Mills et al., 2012). Of note, within-group results appeared robust to publication bias (trim-and-fill, FSN).

Regarding between-group models, in contrast to Roberts et al. (2015), we did not find evidence that trauma-focused treatments were associated with significantly poorer treatment retention relative to either all comparators or the manualized SUD treatments. With the exception of Coffey et al. (2016), none of the studies described having specifically addressed treatment retention for those assigned to a trauma-focused intervention.^7^ Although the reason for the discrepancy is unclear, one possible explanation is that only one of the four RCTs included in the earlier trauma-focused model had an active comparison condition (Sannibale et al., 2013), whereas in the current study eight out of twelve had an active comparison condition. The current retention finding suggests that people with PTSD/SUD may have difficulty remaining in active treatment (i.e., that which is structured and encourages at home practice) regardless of whether the intervention has trauma-focused elements.

In line with Roberts and colleagues, we found that when compared with all types of comparators, trauma-focused treatments showed a small advantage for reductions in PTSD severity at post-treatment assessment (*g* = 0.29), though the advantage did not persist to the longest follow-up and was not robust to publication bias corrections. We also found that trauma-focused treatments did not out-perform all other comparators on SUD outcomes at either immediate or longest follow-up. Although Roberts and colleagues (2015) found an advantage for trauma-focused interventions on PTSD outcomes at both post-treatment and later follow-ups and SUD outcomes at later follow-ups, their group of studies only included comparisons involving no/minimal attention conditions. Although such comparisons are important (e.g. for establishing absolute efficacy; Smith & Glass, 1977), they provide a less rigorous assessment of efficacy and generally should not be used to determine when a particular treatment approach should be recommended relative to other potential therapies (i.e., relative efficacy; Wampold & Imel, 2015).

When we restricted the models to studies involving a manualized SUD treatment, not only did the post-treatment advantage for trauma-focused interventions for PTSD outcomes disappear, but the immediate post-treatment model regarding SUD outcomes indicated a small advantage for manualized SUD treatments (*g* = −0.27). Thus, when compared with trauma-focused interventions, manualized SUD treatments did not differ significantly regarding PTSD outcomes and performed somewhat better on early SUD outcomes, a pattern of findings that held up in our sensitivity analyses including only studies using trauma-focused treatments based on empirically supported PTSD interventions. It is currently not clear how manualized SUD treatments might facilitate such marked improvement in PTSD. Although it remains to be empirically tested in future research, it is possible that the behavioral skills gained in most manualized SUD treatments (e.g., communication and assertiveness, distress tolerance, anger management, contingency planning, reaching out for healthy social support) are helpful in addressing underlying dimensions of psychopathology common to both SUD and PTSD. Specifically, gaining such skills and more consistently availing oneself of adequate social support may mitigate current stress and temper the stress reactivity so common among those with PTSD and SUD (Breese, Sinha, & Heilig, 2011; Lovallo, 2006; Morris, Hellman, Abelson, & Rao, 2016; Steudte-Schmiedgen et al., 2015). Additionally, non-specific factors (e.g., therapeutic alliance, expectancy; Wampold & Imel, 2015) may play a role by increasing hope as well as facilitating stress reduction through contact with a caring treatment provider.

Turning to non-trauma-focused treatments, we found no differences between these treatments and all comparators on SUD or PTSD outcomes at post-treatment or follow-up. Three of the four comparisons with manualized SUD treatments were also non-significant. However, manualized SUD treatments significantly out-performed non-trauma-focused treatments at the longest follow-up on PTSD (*g* = −0.28). The present finding of no differences on PTSD or SUD outcomes for non-trauma-focused interventions relative to all comparators is consistent with the results obtained by Roberts et al. (2015). Importantly, the current study confirms the lack of differences based on a much larger sample of studies. Although neither of the primary models regarding treatment retention for non-trauma-focused interventions were significant, models accounting for possible publication bias suggest that non-trauma-focused interventions may show higher treatment retention relative to all comparators and manualized SUD treatment.

While the between-group models provided some indication of potential treatment differences, none of these effects were robust to publication bias as assessed by FSN. This, coupled with potentially high risk of bias for some studies in some domains (e.g., lack of masked outcome assessors) highlights the tentative nature of these results. Importantly, however, the FSN is designed to guard against acceptance of false positives and not false null results (Rosenthal, 1979). Thus, even if numerous relevant unpublished null trials were added, the overall pattern of no or minimal between-group differences found in the present study likely would not change.

Overall, we found scant evidence that any of the tested moderators significantly influenced outcomes, potentially in part because these tests were underpowered (Valentine et al., 2010). Alongside a majority of null associations, attrition rate was associated with larger effects in three models, group delivery was linked with smaller effects in one model, and percentage of participants reporting drug use was linked with larger effects in one model. The most consistent moderator was study recruitment site. Studies conducted in SUD clinical sites showed substantially larger effects in five within-group models (*g*s = 0.48–1.01) and three between-group models (*g*s = 0.55–1.11), with six of the eight significant outcomes pertaining to SUD. It is possible that patients enrolled through SUD treatment clinics participated in additional SUD programming that was helpful to them and/or they had more severe substance use profiles (see Simpson et al., 2020) and thus had more room for improvement. Additionally, clinicians at SUD treatment clinics likely have more experience treating individuals with SUD (with or without PTSD) than those in university or laboratory settings where community-based recruiting was the norm. Study recruitment site also appears to account for the smaller effects obtained for integrated trauma-focused treatments relative to non-integrated trauma-focused treatments.

The central finding of this meta-analysis of behavioral RCTs for people with PTSD/SUD is that while there were medium to large within-group effects for all who received some form of treatment, between-group effects were generally small and non-significant. Although these findings may be surprising to some and are generally contrary to received wisdom (see Brady, Dansky, Back, Foa, & Carroll, 2001; Brown & Wolfe, 1994), we believe the findings are potentially positive from a public health standpoint.

Consistent with the larger psychotherapy literature (Benish, Imel, & Wampold, 2008; Frost, Laska, & Wampold, 2014; Imel, Wampold, Miller, & Fleming, 2008; Wampold & Imel, 2015; Wampold et al., 1997), the present findings suggest that when people with PTSD and SUD have access to *bona fide* treatments (i.e., with a cogent rationale and intended to be therapeutic; Wampold et al., 1997), they generally improve on both aspects of their comorbidity and further and that the specific type of care may not be especially consequential. Indeed, the finding that manualized SUD treatments were not inferior to trauma-focused nor non-trauma-focused treatments on either PTSD or SUD outcomes and showed modest advantages over both in two between-group models suggests that individuals with this comorbidity may address their mental health challenges with treatments that may be more accessible. Although certain patient characteristics not tested in the current analysis may moderate treatment outcomes (e.g. “treatment matching”), our results encouragingly suggest that adults with PTSD/SUD have *options* with regard to effective PTSD/SUD treatment.

Based on the reviewed studies, it appears that CBT treatments for SUD that include attention to and assessment of current PTSD is likely to confer benefit that is not significantly different from trauma-focused and non-trauma-focused treatments on PTSD outcomes and possibly greater benefit on SUD outcomes. It is, however, important to consider the context for these findings—a set of RCTs wherein both investigators and participants acknowledged the presence and clinical relevance of participants’ comorbid conditions, both conditions were thoroughly assessed over time, and treatment was delivered individually with the support of specific training and supervision. Thus, we do not interpret the current findings to mean that patients with comorbid PTSD/SUD can simply be given standard SUD care with no individualized attention to their PTSD. Nevertheless, patients with PTSD/SUD in addiction treatment settings may be reassured that existing manualized SUD treatments, such as Relapse Prevention (Marlatt & Gordon, 1980), may help them successfully address both their SUD and PTSD in the context of high-fidelity care and ongoing assessment of both PTSD and substance use. Such framing may be more palatable than the old admonitions that patients need to get (and stay) sober before they may be considered stable enough to undertake PTSD treatment (see Brown & Wolfe, 1994, for an overview).

These meta-analytic findings notwithstanding, the epidemiologic patterns cited earlier regarding the high rates of both PTSD and SUD chronicity (Simpson et al., 2020) suggest that much work remains to be done to close the gap between the best practices generally used in RCTs and the quality of care available through community SUD and mental health clinics (see Kilbourne et al., 2018) before the field is in a position to fully deliver on the idea that comorbid patients’ needs will be met no matter where they present for care. Additionally, because there is not yet a sufficient number of trials examining any one of the more promising trauma-focused treatments (see Table A.6 for individual study effect sizes) to test more granular meta-analytic models, we do not yet know whether one (or more) of these specific treatments will eventually prove especially efficacious. The field also has yet to systematically assess treatment acceptability beyond treatment retention and gleaning such information from both treatment completers and non-completers could help clinicians and researchers address the nearly universal low treatment completion rates in the extant relevant literature. Additionally, it would be useful to experimentally evaluate the degree to which patient preference might play a role in outcomes (see Zoellner, Roy-Byrne, Mavissakalian, & Feeny, 2019) and there is almost certainly a great deal to learn about optimizing patient/treatment matching (see Bailey et al., 2019; Hien et al., 2019). Thus, there are a number of interesting and important future research directions to be pursued and we hope that this meta-analysis will provide some useful guideposts.

The current meta-analysis has noteworthy strengths and weaknesses. Strengths include incorporating both relevant unpublished trial results and pertinent subsets of participants from larger published trials. This meta-analysis is better powered than previous ones, which allowed comparisons between trauma-focused or non-trauma-focused treatments and commonly provided CBT treatments for SUD matched on time and attention. Although the number of available studies was likely not sufficient to adequately power moderator tests (Valentine et al., 2010), these exploratory analyses may aid in hypothesis generation as the field collectively works to identify factors that may influence outcomes (e.g., treatment setting, patient factors reflecting need for trauma-focused treatment vs. manualized SUD).

As is commonly the case in meta-analysis, primary weaknesses relate to the meta-analytic sample itself. Although the largest review to date, as noted above, it is likely that some tests (e.g., moderators, between-group models) were underpowered. This is a particularly salient concern for the between-group models in which small effects are to be expected (Baardseth et al., 2013; Goldberg et al., 2018; Wampold & Imel, 2015). The high degree of heterogeneity in many within- and between-group models further reduced statistical power and highlights the tenuous nature of some effect size estimates. We found some evidence of publication bias for the non-trauma-focused treatment retention models, but on the whole, results did not change when accounting for this source of bias in trim-and-fill models. Additionally, many investigators did not use methods that conceal allocation during the randomization process or report whether assessments were masked or random sequence generators were used.

Both treatment completion and assessment completion across most studies was low, which can introduce a host of potential biases even when intention-to-treat analyses are conducted (i.e., biases due to data not missing at random; Graham, 2009). Low treatment and assessment completion suggests that the field has yet to develop treatment options and/or study retention methods that are either appealing or compelling enough to offset the substantial emotional dysregulation (Westphal, Aldao, & Jackson, 2017), social instability (Simpson et al., 2019), and avoidance (Naifeh, Tull, & Gratz, 2012; Simpson, Jakupcak, & Luterek, 2006) common for people with co-occurring PTSD and SUD. Strategies for supporting treatment session attendance have been tested, but trial outcomes suggest that much work remains to be done (see Coffey et al. (2016) described above and Schacht et al., (2017) for an evaluation of contingency management to improve PE session attendance). Clinical researchers could borrow from successful efforts to increase involvement of racial/ethnic minorities in clinical trials using participatory research methods (see McFarlane, Occa, Peng, Awonuga & Morgan, 2021 for a systematic review) such that individuals with lived experience pertaining to PTSD/SUD help develop and refine treatment and trial design to address the persistent problem of sub-optimal treatment and study retention.

Finally, while we opted to conduct traditional meta-analyses in an attempt to replicate and extend the extant literature, future research in this area would benefit from network meta-analyses. Such an approach would enable an evaluation of the relative efficacy of trauma-focused interventions compared to non-trauma-focused interventions given that currently only two studies have made such comparisons (Najavits et al., 2018; Norman et al., 2019).

### 4.1. Conclusions

The current meta-analysis largely affirms the idea that there is “no wrong door” when it comes to treatment options for individuals with PTSD/SUD (see Simpson et al., 2017). Specifically, we found evidence that trauma-focused, non-trauma-focused, manualized SUD treatments, and SUD TAU are all associated with significant improvements on both PTSD and SUD outcomes.^8^ Between-group differences were less consistent and much less robust. Trauma-focused treatments showed slight indications of advantage relative to all comparators regarding PTSD outcomes although manualized SUD treatments also showed slight indications of advantage relative to trauma-focused and non-trauma-focused treatments regarding SUD outcomes. These findings have important public health implications in so far as they suggest that individuals with PTSD/SUD may benefit from relatively readily available (Hendershot, Witkiewitz, George, & Marlatt, 2011; VA/DoD Management of Substance Use Disorders Workgroup, 2015) manualized CBT treatments for SUD rather than requiring specialized options that address both SUD and PTSD with the caveat that measurement based, high quality individual delivery of such care may be necessary to see the degree of improvement evidenced in the present collection of RCTs. The existence of viable treatment options for people with PTSD/SUD lays a solid foundation for future inquiries into optimizing patient-treatment matching and the role of patient preference in recovery, both uncharted but potentially quite fertile territory to explore.

## Supplementary Material

Supplement Simpson et al. (2021) Efficacy and acceptability of interventions for co-occurring PTSD and SUD; A meta-analysis

## Figures and Tables

**Table 1:** Participant-level characteristics by study.

Study	ITT N	Treatment	Comparator	Age	% Female	% Racial/Ethnic Minority	% Met PTSD Diagnostic Criteria	% Met SUD Diagnostic Criteria	% with Current Drug Use

Back et al. (2019)	81	ITF	Manual SUD	40.4	9.9	39.5	100	100	37.1
Boden et al. (2011)	98	NTF	TAU	54	0	81	NA	100	80.6
Brief PC	386	NTF	No/minimal tx	32.15	13.40	22.25	NA	NA	NA
Capone et al. (2014)	44	NTF	TAU	34.29	4.55	15.85	100	100	38.6
Coffey et al. (2016)	126	TF	TAU^1^	34	46	20.6	100	100	NA
van Dam, Ehring, Vedel, and Emmelkamp (2013)	34	TF	Manual SUD	42.3	32.4	26.5	61.8	100	55.9
Foa et al. (2013)	165	TF (+Nal)	Manual SUD	42.7	34.5	69.7	100	100	NA
		TF (+PL)	Manual SUD						
Haller PC	101	ITF	TAU^2^	46.4	10.9	37.6	100	100	58.5
Hamblen (2020)	129	TF	TAU	44.2	5.4	32.8	100	100	92
Hien, Cohen, Miele, Litt, and Capstick (2004)	75	NTF	Manual SUD	36	100	36	88	100	65.3
Hien et al. (2009)	353	NTF	TAU^1^	39.2	100	54.4	80.4	100	91.2
Kehle-Forbes et al. (2018)^3^	183	ITF	TF	44.1	7.75	58.95	100	100	18
McGovern, Lambert-Harris, Alterman, Xie, and Meier (2011)	53	NTF	Manual SUD	37.3	56.6	8.7	100	100	47.2
McGovern et al. (2015)	221	NTF	Manual SUD	35.3	59.3	4.5	100	100	51.1
Mills et al. (2012)	103	ITF	No/minimal tx	33	62.1	5.8	100	100	79.6
Myers, Browne, and Norman (2015)	40	NTF	Manual SUD	42.2	100	48.4	80.6	100	NA
Najavits et al. (2018) ^3^	52	ITF	NTF	48.7	26.9	40	100	100	50
Norman et al. (2019) ^3^	119	ITF	NTF	41.6	10.1	34.5	95.8	100	NA
Perez-Dandieu and Tapia (2014)	12	TF	TAU	29.5	100	NA	100	100	83.3
Possemato et al. (2019) ^3^	30	NTF	NTF	38.6	6.7	20	76.7	100	NA
Ruglass et al. (2017)	110	ITF	Manual SUD	44.4	36.4	81.8	64.5	98.2	66.4
Sannibale et al. (2013)	62	ITF	Manual SUD	41.2	53	NA	100	95	15
Schacht et al. (2017) ^3^	58	TF	TF	37.4	79	29	100	100	100
Schafer et al. (2019)	343	NTF	Manual SUD	40.97	100	9.6	NA	100	NA
Simpson et al. (2021) ^4^	101	TF	Manual SUD	42.1	56	47	100	100	38.6
Stappenbeck et al. (2015)	78	NTF	No/minimal tx	44.3	48.7	57.7	100	100	NA
Vujanovic, Smith, Tipton, and Schmitz (2018) ^4^	41	ITF	Manual SUD	44.9	53.7	75.6	NA	100	NA
Zlotnick, Johnson, and Najavits (2009)	49	NTF	TAU	34.6	100	53.1	83.5	100	NA

Note: ITT N = intention-to-treat sample size; NA = not available; PC = Personal Communication; Manual SUD = manualized SUD treatment; No tx = no/minimal treatment; TAU = SUD TAU; NA = information not collected/reported; NTF = not trauma-focused; TF = trauma-focused; ITF = integrated trauma-focused; NAL = Naloxone; PL = placebo drug.

1 Participants received SUD TAU and a general healthy lifestyle or health-oriented attention placebo control.

2 Participants received an integrated CBT intervention to address depression and SUD.

3 Studies that did not contribute to between-group analyses.

4 Unpublished studies at time analyses undertaken.

**Table 2:** Within-group effects separated by treatment type, domain, and time point.

Treatment	Domain	Time	k	ES	I^2^	k_imp_	ES_adj_	FSN

Trauma	PTSD	post	17	1.11 [0.86, 1.35]	85.37 [73.66, 93.62]	4	0.93 [0.65, 1.21]	3223
Trauma	SUD	post	16	0.82 [0.47, 1.16]	93.55 [88.77, 97.77]	0	0.82 [0.47, 1.16]	1376
Trauma	PTSD	FU	15	1.10 [0.81, 1.39]	87.49 [77.34, 94.85]	0	1.10 [0.81, 1.39]	2305
Trauma	SUD	FU	15	0.97 [0.62, 1.33]	92.98 [87.45, 97.18]	0	0.97 [0.62, 1.33]	2009
Non-trauma	PTSD	post	14	0.65 [0.45, 0.84]	79.67 [62.03, 93.21]	0	0.65 [0.45, 0.84]	1178
Non-trauma	SUD	post	13	0.44 [0.29, 0.59]	72.47 [44.35, 89.02]	5	0.55 [0.41, 0.70]	709
Non-trauma	PTSD	FU	12	0.76 [0.53, 0.99]	78.82 [55.06, 91.35]	0	0.76 [0.53, 0.99]	934
Non-trauma	SUD	FU	12	0.43 [0.20, 0.67]	86.36 [72.03, 95.30]	1	0.48 [0.24, 0.72]	479
Manual SUD	PTSD	post	13	0.85 [0.58, 1.11]	79.26 [56.96, 91.99]	2	0.76 [0.50, 1.02]	800
Manual SUD	SUD	post	13	0.87 [0.41, 1.33]	94.70 [89.48, 98.46]	0	0.87 [0.41, 1.33]	704
Manual SUD	PTSD	FU	11	1.01 [0.70, 1.31]	75.30 [45.91, 92.13]	0	1.01 [0.70, 1.31]	628
Manual SUD	SUD	FU	11	0.80 [0.43, 1.18]	87.61 [73.58, 96.32]	0	0.80 [0.43, 1.18]	469
SUD TAU	PTSD	post	10	0.58 [0.30, 0.86]	85.71 [68.45, 96.28]	1	0.65 [0.35, 0.94]	449
SUD TAU	SUD	post	8	0.30 [0.16, 0.45]	54.15 [26.95, 97.93]	0	0.30 [0.16, 0.45]	108
SUD TAU	PTSD	FU	8	0.79 [0.54, 1.05]	77.46 [47.09, 94.21]	0	0.79 [0.54, 1.05]	481
SUD TAU	SUD	FU	8	0.52 [0.16, 0.88]	91.73 [79.59, 98.25]	0	0.52 [0.16, 0.88]	164
No tx	PTSD	post	5	0.49 [0.27, 0.71]	41.91 [0.00, 92.12]	0	0.49 [0.27, 0.71]	58
No tx	SUD	post	5	0.61 [0.29, 0.92]	75.46 [28.67, 97.13]	0	0.61 [0.29, 0.92]	108
No tx	PTSD	FU	1	1.04 [0.65, 1.43]	NA	NA	NA	10
No tx	SUD	FU	1	1.29 [0.92, 1.66]	NA	NA	NA	16

Note: k = number of treatment or control arms contributing to effect size estimate; ES = effect size in Hedges’ *g* units; I^2^ = heterogeneity; k_imp_ = number of studies imputed to account for funnel plot asymmetry; ES_adj_ = trim-and-fill adjusted effect size; SUD = substance use disorder symptoms; No tx = no treatment control; Trauma = trauma-focused treatment; Manual SUD = manualized SUD treatment; Integ non-trauma = integrated non-trauma-focused treatment; SUD TAU = SUD treatment-as-usual; FU = follow-up; NA = not available due to insufficient studies.

**Table 3:** Between-group effects separated by comparison, domain, and time point.

Comparison	Domain	Time	k	ES	I^2^	k_imp_	ES_adj_	FSN
Trauma v. all	PTSD	post	12	0.29 [0.07, 0.52]	32.95 [0.00, 91.21]	2	0.22 [−0.05, 0.49]	44^a^
Trauma v. all	SUD	post	11	−0.11 [−0.34, 0.11]	25.34 [0.00, 82.18]	1	−0.15 [−0.37, 0.07]	0
Trauma v. all	PTSD	FU	10	−0.01 [−0.24, 0.22]	20.95 [0.00, 83.54]	1	−0.06 [−0.31, 0.19]	0
Trauma v. all	SUD	FU	10	−0.09 [−0.32, 0.14]	21.72 [0.00, 85.28]	2	−0.17 [−0.43, 0.09]	0
Trauma v. Manual SUD	PTSD	post	8	0.15 [−0.06, 0.37]	4.39 [0.00, 88.82]	0	0.15 [−0.06, 0.37]	0
Trauma v. Manual SUD	SUD	post	8	−0.27 [−0.48, −0.06]	0.00 [0.00, 78.83]	0	−0.27 [−0.48, −0.06]	8^a^
Trauma v. Manual SUD	PTSD	FU	7	−0.03 [−0.29, 0.23]	3.06 [0.00, 90.04]	1	−0.11 [−0.43, 0.22]	0
Trauma v. Manual SUD	SUD	FU	7	−0.21 [−0.47, 0.04]	6.06 [0.00, 92.27]	0	−0.21 [−0.47, 0.04]	0
Non-trauma v. all	PTSD	post	10	0.11 [−0.03, 0.26]	4.73 [0.00, 51.86]	0	0.11 [−0.03, 0.26]	0
Non-trauma v. all	SUD	post	10	0.12 [−0.03, 0.28]	27.33 [0.00, 67.01]	0	0.12 [−0.03, 0.28]	5
Non-trauma v. all	PTSD	FU	8	−0.10 [−0.30, 0.09]	12.72 [0.00, 65.79]	1	−0.09 [−0.28, 0.11]	0
Non-trauma v. all	SUD	FU	8	−0.04 [−0.21, 0.13]	16.09 [0.00, 68.85]	0	−0.04 [−0.21, 0.13]	0
Non-trauma v. Manual SUD	PTSD	post	4	0.01 [−0.25, 0.28]	0.00 [0.00, 88.45]	1	−0.02 [−0.28, 0.23]	0
Non-trauma v. Manual SUD	SUD	post	5	0.10 [−0.18, 0.39]	33.30 [0.00, 88.80]	3	−0.11 [−0.40, 0.19]	0
Non-trauma v. Manual SUD	PTSD	FU	4	−0.28 [−0.56, −0.00]	0.00 [0.00, 85.46]	0	−0.28 [−0.56, −0.00]	1
Non-trauma v. Manual SUD	SUD	FU	4	−0.21 [−0.44, 0.02]	0.00 [0.00, 88.80]	1	−0.27 [−0.48, −0.05]	0

Note: k = number of comparisons contributing to effect size estimate; ES = effect size in Hedges’ *g* units; I^2^ = heterogeneity; k_imp_ = number of studies imputed to account for funnel plot asymmetry; ES_adj_ = trim-and-fill adjusted effect size; FSN = fail-safe N with superscripted a for instances in which FSN indicated a significant effect was not robust to publication bias; SUD = substance use disorder symptoms; Non-trauma = integrated non-trauma-focused treatment; Trauma = trauma-focused treatment; v. = versus; Manual SUD = manualized SUD treatment; FU = follow-up.

**Table 4:** Treatment completion for trauma-focused or non-trauma-focused treatments versus comparators.

Comparison	k	OR	I^2^	k_imp_	OR_adj_	k_out_	OR_out_	FSN

Trauma v. all	8	0.85 [0.60, 1.21]	0.00 [0.00, 72.80]	2	0.94 [0.68, 1.31]	0	0.85 [0.60, 1.21]	0
Trauma v. Manual SUD	6	0.92 [0.63, 1.36]	0.00 [0.00, 83.67]	0	0.92 [0.63, 1.36]	0	0.92 [0.63, 1.36]	0
Non-trauma v. all	6	1.22 [0.93, 1.60]	0.00 [0.00, 71.98]	2	1.30 [1.00, 1.68]	0	1.22 [0.93, 1.60]	0
Non-trauma v. Manual SUD	5	1.24 [0.87, 1.78]	0.00 [0.00, 79.37]	2	1.41 [1.01, 1.95]	0	1.24 [0.87, 1.78]	0

Note: k = number of comparisons contributing to effect size estimate; OR = odds ratio, with ORs > 1 indicating higher treatment completion in the experimental group relative to the control condition; I^2^ = heterogeneity; k_imp_ = number of studies imputed to account for funnel plot asymmetry; OR_adj_ = trim-and-fill adjusted effect size; k_out_ = number of outliers detected; OR_out_ = odds ratio estimate with outliers removed; FSN = fail-safe N; Non-trauma = integrated non-trauma-focused treatment; Trauma = trauma-focused treatment; v. = versus; Manual SUD = manualized SUD treatments

## Footnotes

1 This was moot for the non-trauma-focused interventions as they are all integrated.

2 Roberts et al. (2015) included four studies that did not meet the current study inclusion criteria: 1) Coffey, Stasiewicz, Hughes, and Brimo (2006) did not assess substance use post-treatment; 2) Najavits, Gallop, and Weiss (2006) included only adolescents; 3) Frisman, Ford, Lin, Mallon, and Chang (2008) did not include a measure of PTSD symptoms, and 4) when we reached out to Dr. Mueser et al. (2008) to inquire about the possibility of acquiring summary information on the subset of individuals in their sample with current SUD, he informed us that they did not have this information as they queried only lifetime SUD and he also noted that the participants did not necessarily have PTSD.

3 To be considered in the between-group comparisons, studies needed to assign participants to at least two conditions that were evaluating distinctly different conditions rather than testing a core intervention with and without an add-on such as peer support (i.e., Possemato et al., 2019) or contingency management (i.e., Schacht, Brooner, King, Kidorf, & Peirce, 2017), or different sequencing of the same interventions (i.e., Kehle-Forbes et al., 2019).

4 Foa et al. (2013) tested PE while both Back et al. (2018) and Ruglass et al. (2017) tested COPE, which integrates PE and CBT for SUD.

5 These figures pertain only to studies that provided information on diagnostic status.

6 The effect sizes and confidence intervals for these four tests were as follows: PTSD post: 0.24 [−0.15, 0.63]; SUD post: −0.28 [−0.55, −0.01]; PTSD FU: 0.07 [−0.31, 0.44], and SUD FU: −0.01 [−0.47, 0.45].

7 Of note, the Coffey et al. (2016) study found that adding motivational enhancement regarding session attendance to Prolonged Exposure did not lead to statistically different treatment retention relative to those assigned to receive only Prolonged Exposure in the residential rehabilitation setting in which the study took place.

8 Note: The sole study examining outcomes at extended follow-up for those assigned to no/minimal treatment (Mills et al., 2012) also found that those randomized to find their own treatment in the community had strong within person improvements on PTSD and SUD. However because nearly all of those participants reported engaging in SUD care, this condition is more like a SUD treatment as usual comparator than a no/minimal treatment comparator.

## Abbreviations:

AUD: alcohol use disorder
CBT: cognitive behavioral treatment
CPT: cognitive processing therapy
COPE: concurrent treatment of PTSD and substance use disorders using prolonged exposure
DUD: drug use disorder
FSN: fail safe N
ICBT: intent-to-treat
ITT: integrated cognitive behavioral therapy
NIAAA: national institute on alcohol abuse and alcoholism
PTSD: posttraumatic stress disorder
PE: prolonged exposure
RCT: randomized clinical trial
SUD: substance use disorder
VA/DoD: Veterans’ Affairs/Department of Defense.
