# Supplementary material for: Efficacy and acceptability of interventions for co-occurring PTSD and SUD: A meta-analysis
Source: J Anxiety Disord. Author manuscript; Available in PMC 2022 Feb 7. (PMC8819868; doi:10.1016/j.janxdis.2021.102490)
Supplement: Supplement Simpson et al. (2021) Efficacy and acceptability of interventions for co-occurring PTSD and SUD; A meta-analysis [file NIHMS1773329-supplement-Supplement_Simpson_et_al___2021__Efficacy_and_acceptability_of_interventions_for_co-occurring_PTSD_and_SUD__A_meta-analysis.docx]

Table A.1. PRISMA Checklist

| **Section/topic** | **#** | **Checklist item** | **Reported on page #** |
| --- | --- | --- | --- |
| **TITLE** | | |  |
| Title | 1 | Identify the report as a systematic review, meta-analysis, or both. | 1 |
| **ABSTRACT** | | |  |
| Structured summary | 2 | Provide a structured summary including, as applicable: background; objectives; data sources; study eligibility criteria, participants, and interventions; study appraisal and synthesis methods; results; limitations; conclusions and implications of key findings; systematic review registration number. | 2 |
| **INTRODUCTION** | | |  |
| Rationale | 3 | Describe the rationale for the review in the context of what is already known. | 3-7 |
| Objectives | 4 | Provide an explicit statement of questions being addressed with reference to participants, interventions, comparisons, outcomes, and study design (PICOS). | 7-8 |
| **METHODS** | | |  |
| Protocol and registration | 5 | Indicate if a review protocol exists, if and where it can be accessed (e.g., Web address), and, if available, provide registration information including registration number. | 8 |
| Eligibility criteria | 6 | Specify study characteristics (e.g., PICOS, length of follow-up) and report characteristics (e.g., years considered, language, publication status) used as criteria for eligibility, giving rationale. | 8-10 |
| Information sources | 7 | Describe all information sources (e.g., databases with dates of coverage, contact with study authors to identify additional studies) in the search and date last searched. | 10 |
| Search | 8 | Present full electronic search strategy for at least one database, including any limits used, such that it could be repeated. | TA.2 |
| Study selection | 9 | State the process for selecting studies (i.e., screening, eligibility, included in systematic review, and, if applicable, included in the meta-analysis). | 10 |
| Data collection process | 10 | Describe method of data extraction from reports (e.g., piloted forms, independently, in duplicate) and any processes for obtaining and confirming data from investigators. | 10 |
| Data items | 11 | List and define all variables for which data were sought (e.g., PICOS, funding sources) and any assumptions and simplifications made. | 11-12 |
| Risk of bias in individual studies | 12 | Describe methods used for assessing risk of bias of individual studies (including specification of whether this was done at the study or outcome level), and how this information is to be used in any data synthesis. | 12-13 |
| Summary measures | 13 | State the principal summary measures (e.g., risk ratio, difference in means). | 13 |
| Synthesis of results | 14 | Describe the methods of handling data and combining results of studies, if done, including measures of consistency (e.g., I^2^) for each meta-analysis. | 13-15 |
| Risk of bias across studies | 15 | Specify any assessment of risk of bias that may affect the cumulative evidence (e.g., publication bias, selective reporting within studies). | 15 |
| Additional analyses | 16 | Describe methods of additional analyses (e.g., sensitivity or subgroup analyses, meta-regression), if done, indicating which were pre-specified. | 15-16 |
| **RESULTS** | | |  |
| Study selection | 17 | Give numbers of studies screened, assessed for eligibility, and included in the review, with reasons for exclusions at each stage, ideally with a flow diagram. | 16 |
| Study characteristics | 18 | For each study, present characteristics for which data were extracted (e.g., study size, PICOS, follow-up period) and provide the citations. | T1, TA.3 |
| Risk of bias within studies | 19 | Present data on risk of bias of each study and, if available, any outcome level assessment (see item 12). | 17-18, TA.5 |
| Results of individual studies | 20 | For all outcomes considered (benefits or harms), present, for each study: (a) simple summary data for each intervention group (b) effect estimates and confidence intervals, ideally with a forest plot. | TA.6 |
| Synthesis of results | 21 | Present results of each meta-analysis done, including confidence intervals and measures of consistency. | 18-20,  TA.2-4 |
| Risk of bias across studies | 22 | Present results of any assessment of risk of bias across studies (see Item 15). | 20 |
| Additional analysis | 23 | Give results of additional analyses, if done (e.g., sensitivity or subgroup analyses, meta-regression [see Item 16]). | 20-21 |
| **DISCUSSION** | | |  |
| Summary of evidence | 24 | Summarize the main findings including the strength of evidence for each main outcome; consider their relevance to key groups (e.g., healthcare providers, users, and policy makers). | 21-28 |
| Limitations | 25 | Discuss limitations at study and outcome level (e.g., risk of bias), and at review-level (e.g., incomplete retrieval of identified research, reporting bias). | 28-29 |
| Conclusions | 26 | Provide a general interpretation of the results in the context of other evidence, and implications for future research. | 20-30 |
| **FUNDING** | | |  |
| Funding | 27 | Describe sources of funding for the systematic review and other support (e.g., supply of data); role of funders for the systematic review. | 31 |

Table notes: T = Table; TA.X = Appendix Materials Table

Table A.2. Search databases, strategies, and terms. Results were not limited by publication date or language.

| Database (host) | Search Strategy |
| --- | --- |
| PubMed (National Center for Biotechnology Information) | ("Stress Disorders, Post-Traumatic"[Mesh] OR ptsd OR "posttraumatic stress" OR "post traumatic stress" OR "war neurosis" OR shell shock* OR shellshock* OR "combat neurosis")  AND  ("Substance-Related Disorders"[Mesh] OR "Behavior, Addictive"[Mesh] OR alcohol* OR cannabis OR cocaine* OR heroin OR methamphetamine* OR amphetamine* OR "substance use" OR "substance abuse" OR “drug abuse” OR “drug dependence” OR marijuana OR inhalant* OR opiate* OR stimulant* OR addiction)  AND  ("Randomized Controlled Trial" [Publication Type] OR "Randomized Controlled Trials as Topic"[Mesh] OR random* OR "Controlled Clinical Trial" [Publication Type] OR  "Controlled Clinical Trials as Topic"[Mesh] OR (controlled AND examination*) OR (controlled AND study) OR (controlled AND studies) OR (controlled AND trial) OR (controlled AND trials)) |
| Embase (Elsevier) | ('posttraumatic stress disorder'/exp OR 'posttraumatic stress disorder' OR 'ptsd'/exp OR ptsd OR 'posttraumatic stress'/exp OR 'posttraumatic stress' OR 'post traumatic stress'/exp OR 'post traumatic stress' OR 'war neurosis'/exp OR 'war neurosis' OR 'shell shock' OR 'shell shocked' OR shellshock* OR 'combat neurosis')  AND  ('drug dependence'/exp OR 'drug dependence' OR 'addiction'/exp OR 'addiction' OR alcohol* OR 'cannabis'/exp OR cannabis OR cocaine* OR 'heroin'/exp OR heroin OR methamphetamine* OR amphetamine* OR 'substance use'/exp OR 'substance use' OR 'substance abuse'/exp OR 'substance abuse' OR 'drug abuse'/exp OR 'drug abuse' OR 'marijuana'/exp OR marijuana OR inhalant* OR opiate* OR stimulant*)  AND  ('controlled clinical trial'/exp OR 'controlled clinical trial' OR 'controlled clinical trial (topic)'/exp OR 'controlled clinical trial (topic)' OR 'randomized controlled trial'/exp OR 'randomized controlled trial' OR 'randomized controlled trial (topic)'/exp OR 'randomized controlled trial (topic)' OR randomized OR randomised) |
| PsycInfo (Ebsco) | (DE "Posttraumatic Stress Disorder" OR DE "Complex PTSD" OR DE "DESNOS" OR DE "Post-Traumatic Stress" OR ptsd OR “posttraumatic” OR “post traumatic” OR “war neurosis” OR “combat neurosis” OR “shell shock” OR “shell shocked” OR shellshock*)  AND  (DE "Substance Use Disorder" OR DE "Addiction" OR DE "Alcoholism" OR DE "Alcoholic Psychosis" OR DE "Drug Addiction" OR DE "Heroin Addiction" OR DE "Drug Abuse" OR DE "Alcohol Abuse" OR DE "Drug Dependency" OR DE "Inhalant Abuse" OR DE "Polydrug Abuse" OR “substance abuse” OR “drug abuse” OR alcohol* OR cannabis OR cocaine OR heroin OR methamphetamine* OR amphetamine* OR “substance use” OR marijuana OR inhalant* OR opiate* OR stimulant*)  AND  (Random* OR (controlled AND examination*) OR (controlled AND study) OR (controlled AND studies) OR (controlled AND trial) OR (controlled AND trials)) |
| Cochrane Central Register of Controlled Trials (Wiley) | *Title, Abstract, Keywords:* (posttraumatic OR "post traumatic" OR ptsd OR "war neurosis" OR "combat neurosis" OR "shell shock" OR "shell shocked" OR shellshock*)  AND  *Title, Abstract, Keywords:* **(**“substance abuse” OR “drug abuse” OR “drug dependence” OR addiction OR alcohol* OR cannabis OR cocaine OR heroin OR methamphetamine* OR amphetamine* OR “substance use” OR marijuana OR inhalant* OR opiate* OR stimulant*) |
| CINAHL Complete (Ebsco) | (MH "Stress Disorders, Post-Traumatic+" OR ptsd OR “posttraumatic” OR “post traumatic” OR “war neurosis” OR “combat neurosis” OR “shell shock” OR “shell shocked” OR shellshock*)  AND  (MH "Substance Abuse+" OR MH "Substance Use Disorders+" OR “substance abuse” OR “drug abuse” OR “drug dependence” OR addiction OR alcohol* OR cannabis OR cocaine OR heroin OR methamphetamine* OR amphetamine* OR “substance use” OR marijuana OR inhalant* OR opiate* OR stimulant*)  AND  (Random* OR controlled) |
| PTSDpubs (formerly PILOT) (US Department of Veterans Affairs) | (SU.EXACT.EXPLODE("Drug Abuse") OR SU.EXACT("Alcohol Abuse" OR "Amphetamine Abuse" OR "Caffeine Abuse" OR "Cannabis Abuse" OR "Cocaine Abuse" OR "Drug Abuse" OR "Hallucinogen Abuse" OR "Inhalant Abuse" OR "Opioid Abuse" OR "Phencyclidine Abuse" OR "Prescription Drug Abuse" OR "Sedative Abuse") OR AB,TI(addiction OR alcohol* OR “drug abuse” OR “substance abuse” OR “substance use” OR “drug dependence”))  AND (SU.EXACT("Randomized Clinical Trial") OR AB,TI(random* OR (controlled AND trial*) OR (controlled AND study) OR (controlled AND studies))) |
| Web of Science Core Collection (Clarivate Analytics) | *TOPIC*: (ptsd OR “posttraumatic” OR “post traumatic” OR “war neurosis” OR “combat neurosis” OR “shell shock” OR “shell shocked” OR shellshock*)  AND  *TOPIC*: (“substance abuse” OR “drug abuse” OR “drug dependence” OR alcohol* OR addiction OR cannabis OR cocaine OR heroin OR methamphetamine* OR amphetamine* OR “substance use” OR marijuana OR inhalant* OR opiate* OR stimulant*)  AND  *TOPIC*: (random* OR (controlled AND trial*) OR (controlled AND study) OR (controlled AND studies))  *Refined by: DOCUMENT TYPES***:** (PROCEEDINGS PAPER OR BOOK CHAPTER OR MEETING ABSTRACT) |
| ProQuest Dissertations and Theses Global (ProQuest) | *Anywhere except full text (ALL):* (ptsd OR “posttraumatic” OR “post traumatic” OR “war neurosis” OR “combat neurosis” OR “shell shock” OR “shell shocked” OR shellshock*)  *Anywhere except full text (ALL):* (“substance abuse” OR “substance use” OR “drug abuse” OR “drug dependence” OR alcohol* OR addiction OR cannabis OR cocaine OR heroin OR methamphetamine* OR amphetamine* OR “substance use” OR marijuana OR inhalant* OR opiate* OR stimulant*)  AND  *Anywhere except full text (ALL):* (random* OR controlled) |
| ClinicalTrials.gov (National Library of Medicine) | *Condition/Disease*: (ptsd OR posttraumatic OR “post traumatic”)  AND  *Other Terms*: (substance OR “drug abuse” OR alcohol OR alcoholism OR cannabis OR cocaine OR heroin OR methamphetamine OR marijuana OR inhalants OR opiates OR stimulants OR “drug use” OR addiction OR AUD OR SUD) AND (random OR randomized OR controlled) |
| International Clinical Trials Registry Platform (World Health Organization) | (drug abuse OR alcohol OR alcoholism OR substance abuse OR addiction OR drug use OR aud OR sud OR substance use OR cannabis OR cocaine OR heroin OR methamphetamine OR methamphetamines OR marijuana OR inhalants OR opiates OR stimulants) *IN THE TITLE*  AND  (posttraumatic stress OR post-traumatic stress OR ptsd) *IN THE CONDITION* |

Table A.3. Treatment-level characteristics by study

| Study | Condition Name | Exper | ITT n | #  wks | | # sess | | # wks req | # sess req | % comp (wks) | % comp (sess) | Modality |
| --- | --- | --- | --- | --- | --- | --- | --- | --- | --- | --- | --- | --- |
| Back 2019 | Concurrent Treatment of PTSD and SUD using Prolonged Exposure (COPE) | yes | 54 | NA | | 12 | | NA | 8 | NA | 66.7 | Individual |
| Back 2019 | Relapse Prevention | no | 27 | NA | | 12 | | NA | 8 | NA | 55.6 | Individual |
| Boden 2011 | Seeking Safety | yes | 59 | 12 | | 36 | | NA | NA | NA | NA | Group |
| Boden 2011 | SUD Treatment As Usual (TAU) | no | 58 | 12 | | 24 | | NA | NA | NA | NA | Group |
| Brief 2013 | VetChange | yes | 265 | 8 | 8 | | 4 | | 4 | NA | 57 | Web-based |
| Brief 2013 | Waitlist (delayed treatment) | no | 121 | NA | | 8 | | NA | 4 | NA | 35.5 | NA |
| Capone 2018 | Integrated Cognitive Behavioral Therapy (ICBT) +TAU | yes | 21 | NA | | 12 | | NA | 8 | NA | 38.1 | Individual & group |
| Capone 2018 | SUD TAU | no | 23 | NA | | NA | | NA | NA | NA | NA | Individual & group |
| Coffey 2016 | Prolonged Exposure (PE) | yes | 45 | 8 | | 12 | | NA | 8 | NA | 62.6 | Individual |
| Coffey 2016 | PE + Motivational Enhancement for PE | yes | 40 | 8 | | 12 | | NA | 8 | NA | 60 | Individual |
| Coffey 2016 | Healthy Lifestyle Education | no | 41 | 8 | | 12 | | NA | 8 | NA | 69 | Individual |
| Foa 2013 | PE, Naltrexone & Supportive Counseling (SC; for alcohol) | yes | 40 | 18 | | 36 | | 18 | NA | 65 | NA | Individual |
| Foa 2013 | PE, Placebo, & SC | yes | 40 | 18 | | 36 | | 18 | NA | 62 | NA | Individual |
| Foa 2013 | Naltrexone & SC | no | 42 | 18 | | 18 | | 18 | NA | 69 | NA | Individual |
| Foa 2013 | Placebo & SC | no | 43 | 18 | | 18 | | 18 | NA | 74 | NA | Individual |
| Haller 2016 | Cognitive Processing Therapy (CPT) + Relapse Prevention | yes | 51 | NA | | 12 | | NA | 6 | NA | 56.9 | Individual & group |
| Haller 2016 | Integrated Cognitive Behavioral Therapy (for comorbid depression & SUD; ICBTd) | no | 50 | NA | | 12 | | NA | 6 | NA | 64 | Individual & group |
| Hamblen 2020 | CBT for PTSD | yes | 64 | 12 | | 12 | | 8 | 8 | 55.3 | 55.3 | Individual & group |
| Hamblen 2020 | TAU -- VA ATC & SUD residential | no | 65 | NA | | NA | | NA | NA | NA | NA | Individual & group |
| Hien 2004 | Seeking Safety | yes | 41 | 12 | | 24 | | NA | 6 | NA | 61 | Individual |
| Hien 2004 | Relapse Prevention | no | 34 | 12 | | 24 | | NA | 6 | NA | 70.6 | Individual |
| Hien 2009 | Seeking Safety | yes | 176 | 6 | | 12 | | NA | 6 | NA | 58.5 | Group |
| Hien 2009 | Women's Health Education | no | 177 | 6 | | 12 | | NA | 6 | NA | 54.2 | Group |
| Kehle-Forbes 2018 | Integrated Motivational Enhancement (MET for alcohol)/PE | yes | 95 | NA | | 16 | | NA | 12 | NA | 23.2 | Individual |
| Kehle-Forbes 2018 | Sequential MET/PE | yes | 88 | NA | | 16 | | NA | 12 | NA | 36.4 | Individual |
| McGovern 2011 | Integrated Cognitive Behavioral Therapy (ICBT) | yes | 32 | 12 | | 12 | | NA | 8 | NA | 50 | Individual |
| McGovern 2011 | Individual addiction counseling (IAC) | no | 21 | 10 | | 10 | | NA | 8 | NA | 47.6 | Individual |
| McGovern 2015 | ICBT | yes | 73 | 12 | | 12 | | NA | 8 | NA | 45.2 | Individual |
| McGovern 2015 | IAC | no | 75 | 12 | | 12 | | NA | 8 | NA | 36 | Individual |
| McGovern 2015 | Standard SUD Care (TAU) | no | 73 | NA | | NA | | NA | NA | NA | NA | Individual |
| Mills 2012 | COPE | yes | 55 | 13 | | 13 | | NA | 5 | NA | 54.5 | Individual |
| Mills 2012 | Usual care (found by participants in community) | no | 48 | 13 | | 13 | | NA | NA | NA | NA | NA |
| Myers 2015 | Seeking Safety + Cognitive Therapy for Battered Women | yes | 31 | 12 | | 25 | | NA | 6 | NA | 45.2 | Individual |
| Myers 2015 | 12-Step Facilitation | no | 9 | 12 | | 25 | | NA | 6 | NA | 44.4 | Individual |
| Najavits 2018 | Creating Change | yes | 26 | NA | | 17 | | NA | NA | NA | NA | Individual |
| Najavits 2018 | Seeking Safety | yes | 26 | NA | | 17 | | NA | NA | NA | NA | Individual |
| Norman 2019 | COPE | yes | 63 | NA | | 12 | | NA | 12 | NA | 31.7 | Individual |
| Norman 2019 | Seeking Safety | yes | 56 | NA | | 12 | | NA | 12 | NA | 66.1 | Individual |
| Perez-Dandieu 2014 | Eye Movement Desensitization and Reprocessing (EMDR) | yes | 6 | 26 | | 8 | | NA | 8 | NA | 100 | Individual |
| Perez-Dandieu 2014 | SUD TAU | no | 6 | NA | | NA | | NA | NA | NA | NA | Individual |
| Possemato 2019 | Web CBT + Peer Support | yes | 15 | NA | | 12 | | NA | 12 | NA | 40 | Web-based & Individual |
| Possemato 2019 | Web CBT | no | 15 | NA | | 12 | | NA | 12 | NA | 60 | Web-based |
| Ruglass 2017 | COPE | yes | 39 | NA | | 12 | | NA | NA | NA | 51.3 | Individual |
| Ruglass 2017 | Relapse Prevention | no | 43 | NA | | 12 | | NA | NA | NA | 46.5 | Individual |
| Ruglass 2017 | Weekly in person monitoring with study research assistant | no | 28 | NA | | 12 | | NA | NA | NA | 85.7 | Individual |
| Sannibale 2013 | Integrated Treatment (IT; includes trauma exposure) | yes | 33 | 12 | | 12 | | NA | 9 | NA | 60.6 | Individual |
| Sannibale 2013 | Alcohol Support | no | 29 | 12 | | 12 | | NA | 9 | NA | 62.1 | Individual |
| Schacht 2017 | PE + Contingency Management for attendance | yes | 28 | NA | | 12 | | NA | 9 | NA | 36 | Individual |
| Schacht 2017 | PE only | no | 30 | NA | | 12 | | NA | 9 | NA | 3 | Individual |
| Schaefer 2019 | Seeking Safety | yes | 111 | NA | | 14 | | NA | 8 | NA | 36.9 | Group |
| Schaefer 2019 | Relapse Prevention | no | 115 | NA | | 14 | | NA | 8 | NA | 28.7 | Group |
| Schaefer 2019 | SUD TAU | no | 117 | NA | | NA | | NA | NA | NA | NA | Group |
| Simpson 2021^1^ | CPT | yes | 41 | NA | | 12 | | NA | 9 | NA | 53.7 | Individual |
| Simpson 2021^1^ | Relapse Prevention | no | 38 | NA | | 12 | | NA | 9 | NA | 60.5 | Individual |
| Simpson 2021^1^ | Assessment only (with minimal telephone support) | no | 22 | NA | | 6 | | NA | 4 | NA | NA | Individual |
| Stappenbeck 2015 | Experiential Acceptance | yes | 27 | NA | | NA | | NA | NA | NA | NA | Individual |
| Stappenbeck 2015 | Cognitive Restructuring | yes | 31 | NA | | NA | | NA | NA | NA | NA | Individual |
| Stappenbeck 2015 | Placebo Attention Control | no | 20 | NA | | NA | | NA | NA | NA | NA | Individual |
| van Dam 2013 | Structured Writing Therapy | yes | 19 | NA | | 30 | | NA | 22 | NA | 52.6 | Individual |
| van Dam 2013 | SUD TAU^2^ | no | 15 | NA | | 20 | | NA | 15 | NA | 73.3 | Individual |
| Vujanovic 2018^1^ | Treatment of Integrated Posttraumatic Stress and Substance Use (TIPSS, includes CPT) | yes | 19 | 6 | | 12 | | NA | 12 | NA | 68.42 | Individual |
| Vujanovic 2018^1^ | CBT for SUD | no | 22 | 6 | | 12 | | NA | 12 | NA | 86.36 | Individual |
| Zlotnick 2009 | Seeking Safety (incarcerated) | yes | 27 | 8 | | 36 | | NA | NA | NA | NA | Group & Individual |
| Zlotnick 2009 | SUD TAU (incarcerated) | no | 22 | 8 | | NA | | NA | NA | NA | NA | Group |

Note: Exper = experimental condition; ITT n = intention-to-treat sample size; # sess = number of sessions; # weeks req = # of weeks required to be considered treatment completer; # sess req = # of sessions required to be considered treatment completer; % comp (wks) = percentage of participants who completed the required number of weeks; % comp (sess) = percentage of participants who completed the required number of sessions; SUD = substance use disorder; PC = personal communication; NA = not available.

^1^Studies that were unpublished when the analyses were conducted.

^2^The van Dam comparison group was labeled SUD TAU but it is apparent from the description of the intervention that it was based on empirically validated CBT for SUD with appropriate manuals referenced.

Table A.4. Treatment and control conditions

| Study | Treatment/Control Name | Treatment/Control Type |
| --- | --- | --- |
| Back 2019 | Concurrent Treatment of PTSD and SUD using Prolonged Exposure (COPE) | Trauma-focused |
| Back 2019 | Relapse Prevention | Manualized SUD |
| Boden 2011 | Seeking Safety | Non-trauma-focused |
| Boden 2011 | SUD TAU | SUD TAU |
| Brief 2013 | VetChange | Non-trauma-focused |
| Brief 2013 | Waitlist | No/minimal treatment |
| Capone 2018 | Integrated Cognitive Behavioral Therapy (ICBT) + SUD TAU | Non-trauma-focused |
| Capone 2018 | SUD TAU | SUD TAU |
| Coffey 2016 | Prolonged Exposure + SUD TAU | Trauma-focused |
| Coffey 2016 | Prolonged Exposure + Motivational Enhancement Therapy (attendance) + SUD TAU | Trauma-focused |
| Coffey 2016 | Healthy Lifestyles + SUD TAU | SUD TAU |
| Foa 2013a | Prolonged Exposure + Naltrexone + Supportive Counseling (alcohol CBT) | Trauma-focused |
| Foa 2013a | Naltrexone + Supportive Counseling (alcohol CBT) | Manualized SUD |
| Foa 2013b | Prolonged Exposure + Placebo + Supportive Counseling (alcohol CBT) | Trauma-focused |
| Foa 2013b | Placebo + Supportive Counseling (alcohol CBT) | Manualized SUD |
| Haller 2016 | Cognitive Processing Therapy (CPT) + Relapse Prevention | Trauma-focused |
| Haller 2016 | Integrated Cognitive Behavioral Therapy (for depression & SUD; ICBTd) | Manualized SUD |
| Hamblen 2020 | CBT for PTSD | Trauma-focused |
| Hamblen 2020 | TAU -- VA ATC & SUD residential | SUD TAU |
| Hien 2004 | Seeking Safety | Non-trauma-focused |
| Hien 2004 | Relapse Prevention | Manualized SUD |
| Hien 2009 | Seeking Safety + SUD TAU | Non-trauma-focused |
| Hien 2009 | Women's Health Education + SUD TAU | SUD TAU |
| Kehle-Forbes 2019 | Integrated Motivational Enhancement (MET for alcohol)/Prolonged Exposure | Trauma-focused |
| Kehle-Forbes 2019 | Sequential Motivational Enhancement (MET for alcohol)/Prolonged Exposure | Trauma focused |
| McGovern 2011 | Integrated Cognitive Behavioral Therapy (ICBT) | Non-trauma-focused |
| McGovern 2011 | Individual addiction counseling (IAC) | Manualized SUD |
| McGovern 2015 | Integrated Cognitive Behavioral Therapy (ICBT) | Non-trauma-focused |
| McGovern 2015 | Individual addiction counseling (IAC) | Manualized SUD |
| McGovern 2015 | Standard SUD Care | SUD TAU |
| Mills 2012 | Concurrent Treatment of PTSD and SUD using Prolonged Exposure (COPE) | Trauma-focused |
| Mills 2012 | Usual care in community | No/minimal treatment |
| Myers 2015 | Seeking Safety + Cognitive Therapy for Battered Women | Non-trauma-focused |
| Myers 2015 | 12-Step Facilitation | Manualized SUD |
| Najavits 2018 | Creating Change | Trauma-focused |
| Najavits 2018 | Seeking Safety | Non-trauma-focused |
| Norman 2019 | Concurrent Treatment of PTSD and SUD using Prolonged Exposure (COPE) | Trauma-focused |
| Norman 2019 | Seeking Safety | Non-trauma-focused |
| Perez-Dandieu 2014 | Eye Movement Desensitization and Reprocessing | Trauma-focused |
| Perez-Dandieu 2014 | SUD TAU | SUD TAU |
| Possemato 2019 | Web-based CBT for PTSD/SUD + Peer Support | Non-trauma-focused |
| Possemato 2019 | Web-based CBT for PTSD/SUD | Non-trauma-focused |
| Ruglass 2017 | Concurrent Treatment of PTSD and SUD using Prolonged Exposure (COPE) | Trauma-focused |
| Ruglass 2017 | Relapse Prevention | Manualized SUD |
| Ruglass 2017 | Symptom monitoring | No/minimal treatment |
| Sannibale 2013 | Integrated exposure-based CBT for PTSD and AUD (IT) | Trauma-focused |
| Sannibale 2013 | CBT for AUD plus supportive counseling (AS) | Manualized SUD |
| Schacht 2017 | Prolonged Exposure + Contingency Management (attendance) | Trauma-focused |
| Schacht 2017 | Prolonged Exposure | Trauma-focused |
| Schaefer 2019 | Seeking Safety | Non-trauma-focused |
| Schaefer 2019 | Relapse Prevention | Manualized SUD |
| Schaefer 2019 | SUD TAU | SUD TAU |
| Simpson 2021^1^ | Cognitive Processing Therapy | Trauma focused |
| Simpson 2021^1^ | Relapse Prevention | Manualized SUD |
| Simpson 2021^1^ | Assessment only (with telephone support) | No/minimal treatment |
| Stappenbeck 2015 | Experiential Acceptance | Non-trauma-focused |
| Stappenbeck 2015 | Cognitive Restructuring | Non-trauma-focused |
| Stappenbeck 2015 | Nutrition Control (attention placebo) | No/minimal treatment |
| van Dam 2013 | Structured Writing Therapy for PTSD | Trauma-focused |
| van Dam 2013 | SUD TAU | SUD TAU |
| Vujanovic 2018^1^ | Treatment of Integrated Posttraumatic Stress and Substance Use (TIPPS; includes Cognitive Processing Therapy) | Trauma-focused |
| Vujanovic 2018^1^ | Cognitive-behavioral therapy for SUD | Manualized SUD |
| Zlotnick 2009 | Seeking Safety | Non-trauma-focused |
| Zlotnick 2009 | SUD TAU | SUD TAU |

^1^Studies that were unpublished when the analyses were conducted.

Table A.5. Cochrane risk of bias ratings by study

| Study | Random Sequence  Generation | Allocation Concealed | Masked Outcome | Attrition Bias | Selective Reporting | Other Bias |  |
| --- | --- | --- | --- | --- | --- | --- | --- |
| Back 2019 | low | high | low | low | low | low | |
| Boden 2011 | low | low | low | low | low | low | |
| Brief 2013 | high | high | high | low | low | low | |
| Capone 2018 | unclear | high | unclear | low | low | low | |
| Coffey 2016 | low | low | low | low | low | low | |
| Foa 2013 | unclear | high | low | low | low | low | |
| Haller 2016 | unclear | high | high | low | high | low | |
| Hamblen 2020 | low | low | low | low | high | high | |
| Hein 2004 | unclear | unclear | unclear | low | high | low | |
| Hein 2009 | low | low | low | low | low | low | |
| Kehle-Forbes 2019 | low | low | low | low | low | low | |
| McGovern 2011 | unclear | high | high | low | low | low | |
| McGovern 2015 | low | high | low | low | low | low | |
| Mills 2012 | low | low | unclear | low | high | low | |
| Myers 2015 | unclear | high | unclear | high | low | unclear | |
| Najavits 2018 | low | high | low | low | low | low | |
| Norman 2019 | low | low | low | low | low | low | |
| Perez-Dandieu 2014 | unclear | high | high | high | low | low | |
| Possemato 2019 | low | high | high | high | low | low | |
| Ruglass 2017 | low | low | low | low | low | high | |
| Sannibale 2013 | low | low | low | low | low | low | |
| Schacht 2017 | low | low | unclear | low | high | low | |
| Schaefer 2019 | low | low | low | low | low | low | |
| Simpson 2021^1^ | low | low | low | low | low | low | |
| Stappenbeck 2015 | unclear | high | unclear | low | high | low | |
| van Dam 2013 | low | low | unclear | low | high | high | |
| Vujanovic 2018^1^ | low | low | low | low | low | low | |
| Zlotnick 2009 | high | high | high | high | low | low | |

Note: low = low risk of bias; unclear = unclear risk of bias; high = high risk of bias; PC = personal communication.

^1^Studies that were unpublished when the analyses were conducted.

Table A.6. Study-level aggregate effect sizes and variances by treatment or control condition^a^

| Study | Domain | Comparison | Time point | ES | Variance | |
| --- | --- | --- | --- | --- | --- | --- |
| Ruglass 2017 Monitoring | PTSD | No/minimal treatment | post | 0.25 | 0.05 |  |
| Brief 2013 WL | PTSD | No/minimal treatment | post | 0.34 | 0.01 |  |
| Stappenbeck 2015 NC | PTSD | No/minimal treatment | post | 0.43 | 0.06 |  |
| Simpson-Kaysen 2019 AO | PTSD | No/minimal treatment | post | 0.61 | 0.05 |  |
| Mills 2012 UC in community | PTSD | No/minimal treatment | post | 0.84 | 0.03 |  |
| Haller 2016 CPT | PTSD | TF | post | 0.23 | 0.02 |  |
| Najavits 2018 Creating Change | PTSD | TF | post | 0.29 | 0.05 |  |
| Hamblen 2020 CBT for PTSD | PTSD | TF | post | 0.51 | 0.03 |  |
| Vujanovic 2018 TIPSS | PTSD | TF | post | 0.7 | 0.06 |  |
| Schacht 2017 PE | PTSD | TF | post | 0.72 | 0.04 |  |
| Kehle-Forbes 2018 Integrated MET/PE | PTSD | TF | post | 0.72 | 0.01 |  |
| Kehle-Forbes 2018 Sequential MET/PE | PTSD | TF | post | 0.86 | 0.01 |  |
| Ruglass 2017 COPE | PTSD | TF | post | 0.89 | 0.07 |  |
| Sannibale 2013 IT | PTSD | TF | post | 0.93 | 0.04 |  |
| Schacht 2017 PE+CM | PTSD | TF | post | 1.09 | 0.07 |  |
| Mills 2012 COPE | PTSD | TF | post | 1.11 | 0.04 |  |
| van Dam 2013 SWT | PTSD | TF | post | 1.12 | 0.11 |  |
| Norman 2019 PE | PTSD | TF | post | 1.36 | 0.05 |  |
| Foa 2013b PE+PL+SC | PTSD | TF | post | 1.39 | 0.08 |  |
| Simpson-Kaysen 2019 CPT | PTSD | TF | post | 1.39 | 0.05 |  |
| Coffey 2016 PE | PTSD | TF | post | 1.85 | 0.08 |  |
| Perez-Dandieu 2014 EMDR | PTSD | TF | post | 1.92 | 0.43 |  |
| Coffey 2016 PE+MET | PTSD | TF | post | 1.95 | 0.08 |  |
| Foa 2013a PE+NAL+SC | PTSD | TF | post | 1.98 | 0.13 |  |
| Back 2019 COPE | PTSD | TF | post | 2.2 | 0.09 |  |
| Hien 2004 Relapse Prevention | PTSD | Manualized SUD | post | 0.05 | 0.04 |  |
| Haller 2016 ICBT | PTSD | Manualized SUD | post | 0.22 | 0.02 |  |
| Schaefer grant RP | PTSD | Manualized SUD | post | 0.3 | 0.02 |  |
| Sannibale 2013 AS | PTSD | Manualized SUD | post | 0.73 | 0.04 |  |
| Myers 2015 12SF | PTSD | Manualized SUD | post | 0.79 | 0.21 |  |
| Vujanovic 2018 CBT | PTSD | Manualized SUD | post | 0.86 | 0.05 |  |
| Foa 2013a NAL+SC | PTSD | Manualized SUD | post | 1.05 | 0.07 |  |
| Ruglass 2017 RP | PTSD | Manualized SUD | post | 1.11 | 0.06 |  |
| McGovern 2015 IAC | PTSD | Manualized SUD | post | 1.12 | 0.03 |  |
| Simpson-Kaysen 2019 RP | PTSD | Manualized SUD | post | 1.14 | 0.04 |  |
| Back 2019 RP | PTSD | Manualized SUD | post | 1.27 | 0.09 |  |
| McGovern 2011 IAC | PTSD | Manualized SUD | post | 1.29 | 0.17 |  |
| Foa 2013b PL+SC | PTSD | Manualized SUD | post | 1.57 | 0.08 |  |
| Hien 2004 Seeking Safety | PTSD | NTF | post | 0.19 | 0.04 |  |
| Schaefer grant Seeking Safety | PTSD | NTF | post | 0.23 | 0.01 |  |
| Najavits 2018 Seeking Safety | PTSD | NTF | post | 0.26 | 0.04 |  |
| Boden 2011 Seeking Safety | PTSD | NTF | post | 0.29 | 0.02 |  |
| Stappenbeck 2015 EA | PTSD | NTF | post | 0.32 | 0.04 |  |
| Possemato 2018 Web CBT + Peer | PTSD | NTF | post | 0.45 | 0.11 |  |
| Stappenbeck 2015 CR | PTSD | NTF | post | 0.46 | 0.04 |  |
| Myers 2015 SS+ | PTSD | NTF | post | 0.62 | 0.08 |  |
| Zlotnick 2009 SS | PTSD | NTF | post | 0.69 | 0.03 |  |
| Brief 2013 VetChange | PTSD | NTF | post | 0.7 | 0.01 |  |
| Norman 2019 SS | PTSD | NTF | post | 0.71 | 0.03 |  |
| Possemato 2018 Web CBT | PTSD | NTF | post | 0.73 | 0.1 |  |
| Capone 2018 ICBT + TAU | PTSD | NTF | post | 0.88 | 0.07 |  |
| Hien 2009 Seeking Safety | PTSD | NTF | post | 1.13 | 0.01 |  |
| McGovern 2015 ICBT | PTSD | NTF | post | 1.38 | 0.03 |  |
| McGovern 2011 ICBT | PTSD | NTF | post | 1.89 | 0.2 |  |
| Perez-Dandieu 2014 TAU (SUD) | PTSD | SUD TAU | post | -0.27 | 0.12 |  |
| Schaefer grant TAU | PTSD | SUD TAU | post | 0.28 | 0.01 |  |
| Boden 2011 TAU (SUD) | PTSD | SUD TAU | post | 0.29 | 0.02 |  |
| Hamblen 2020 TAU | PTSD | SUD TAU | post | 0.34 | 0.02 |  |
| van Dam 2013 SUD TAU | PTSD | SUD TAU | post | 0.37 | 0.07 |  |
| Capone 2018 TAU | PTSD | SUD TAU | post | 0.42 | 0.05 |  |
| Zlotnick 2009 TAU (SUD) | PTSD | SUD TAU | post | 0.65 | 0.04 |  |
| McGovern 2015 SC | PTSD | SUD TAU | post | 1.01 | 0.03 |  |
| Hien 2009 Women's Health ED | PTSD | SUD TAU | post | 1.13 | 0.01 |  |
| Coffey 2016 HLS | PTSD | SUD TAU | post | 1.33 | 0.05 |  |
| Ruglass 2017 COPE | PTSD | TF v. all | post | -0.22 | 0.13 |  |
| Foa 2013b PE+PL+SC | PTSD | TF v. all | post | -0.17 | 0.16 |  |
| Vujanovic 2018 TIPSS | PTSD | TF v. all | post | -0.16 | 0.12 |  |
| Haller 2016 CPT | PTSD | TF v. all | post | 0.01 | 0.04 |  |
| Sannibale 2013 IT | PTSD | TF v. all | post | 0.09 | 0.07 |  |
| Hamblen 2020 CBT for PTSD | PTSD | TF v. all | post | 0.18 | 0.05 |  |
| Simpson-Kaysen 2019 CPT | PTSD | TF v. all | post | 0.34 | 0.06 |  |
| Coffey 2016 PE and PE+MET | PTSD | TF v. all | post | 0.58 | 0.09 |  |
| van Dam 2013 SWT | PTSD | TF v. all | post | 0.83 | 0.16 |  |
| Back 2019 COPE | PTSD | TF v. all | post | 0.93 | 0.18 |  |
| Foa 2013a PE+NAL+SC | PTSD | TF v. all | post | 0.93 | 0.2 |  |
| Perez-Dandieu 2014 EMDR | PTSD | TF v. all | post | 2.19 | 0.55 |  |
| Ruglass 2017 COPE | PTSD | TF v. Manualized SUD | post | -0.22 | 0.13 |  |
| Foa 2013b PE+PL+SC | PTSD | TF v. Manualized SUD | post | -0.17 | 0.16 |  |
| Vujanovic 2018 TIPSS | PTSD | TF v. Manualized SUD | post | -0.16 | 0.12 |  |
| Haller 2016 CPT | PTSD | TF v. Manualized SUD | post | 0.01 | 0.04 |  |
| Sannibale 2013 IT | PTSD | TF v. Manualized SUD | post | 0.09 | 0.07 |  |
| Simpson-Kaysen 2019 CPT | PTSD | TF v. Manualized SUD | post | 0.34 | 0.06 |  |
| Back 2019 COPE | PTSD | TF v. Manualized SUD | post | 0.93 | 0.18 |  |
| Foa 2013a PE+NAL+SC | PTSD | TF v. Manualized SUD | post | 0.93 | 0.2 |  |
| Myers 2015 SS+ | PTSD | NTF v. all | post | -0.17 | 0.29 |  |
| Schaefer grant Seeking Safety | PTSD | NTF v. all | post | -0.07 | 0.03 |  |
| Boden 2011 Seeking Safety | PTSD | NTF v. all | post | 0 | 0.05 |  |
| Hien 2009 Seeking Safety | PTSD | NTF v. all | post | 0 | 0.02 |  |
| Stappenbeck 2015 CR | PTSD | NTF v. all | post | 0.03 | 0.1 |  |
| Zlotnick 2009 SS | PTSD | NTF v. all | post | 0.09 | 0.06 |  |
| Hien 2004 Seeking Safety | PTSD | NTF v. all | post | 0.14 | 0.08 |  |
| Brief 2013 VetChange | PTSD | NTF v. all | post | 0.36 | 0.02 |  |
| McGovern 2011 ICBT | PTSD | NTF v. all | post | 0.43 | 0.22 |  |
| Capone 2018 ICBT + TAU | PTSD | NTF v. all | post | 0.46 | 0.12 |  |
| Myers 2015 SS+ | PTSD | NTF v. Manualized SUD | post | -0.17 | 0.29 |  |
| Schaefer grant Seeking Safety | PTSD | NTF v. Manualized SUD | post | -0.07 | 0.03 |  |
| Hien 2004 Seeking Safety | PTSD | NTF v. Manualized SUD | post | 0.14 | 0.08 |  |
| McGovern 2011 ICBT | PTSD | NTF v. Manualized SUD | post | 0.43 | 0.22 |  |
| Mills 2012 UC in community | PTSD | No/minimal treatment | FU | 1.04 | 0.04 |  |
| Najavits 2018 Creating Change | PTSD | TF | FU | 0.28 | 0.08 |  |
| Haller 2016 CPT | PTSD | TF | FU | 0.28 | 0.03 |  |
| Hamblen 2020 CBT for PTSD | PTSD | TF | FU | 0.38 | 0.03 |  |
| Kehle-Forbes 2018 Integrated MET/PE | PTSD | TF | FU | 0.4 | 0.01 |  |
| van Dam 2013 SWT | PTSD | TF | FU | 0.54 | 0.07 |  |
| Schacht 2017 PE | PTSD | TF | FU | 0.7 | 0.05 |  |
| Kehle-Forbes 2018 Sequential MET/PE | PTSD | TF | FU | 0.73 | 0.02 |  |
| Sannibale 2013 IT | PTSD | TF | FU | 0.83 | 0.04 |  |
| Simpson-Kaysen 2019 CPT | PTSD | TF | FU | 1.15 | 0.06 |  |
| Schacht 2017 PE+CM | PTSD | TF | FU | 1.3 | 0.08 |  |
| Ruglass 2017 COPE | PTSD | TF | FU | 1.36 | 0.07 |  |
| Back 2019 COPE | PTSD | TF | FU | 1.49 | 0.08 |  |
| Norman 2019 PE | PTSD | TF | FU | 1.59 | 0.07 |  |
| Foa 2013b PE+PL+SC | PTSD | TF | FU | 1.6 | 0.1 |  |
| Mills 2012 COPE | PTSD | TF | FU | 1.68 | 0.06 |  |
| Coffey 2016 PE | PTSD | TF | FU | 1.82 | 0.07 |  |
| Coffey 2016 PE+MET | PTSD | TF | FU | 1.96 | 0.09 |  |
| Foa 2013a PE+NAL+SC | PTSD | TF | FU | 2.47 | 0.18 |  |
| Hien 2004 Relapse Prevention | PTSD | Manualized SUD | FU | 0.15 | 0.05 |  |
| Schaefer grant RP | PTSD | Manualized SUD | FU | 0.66 | 0.02 |  |
| Haller 2016 ICBT | PTSD | Manualized SUD | FU | 0.69 | 0.05 |  |
| Myers 2015 12SF | PTSD | Manualized SUD | FU | 0.75 | 0.18 |  |
| Sannibale 2013 AS | PTSD | Manualized SUD | FU | 0.85 | 0.05 |  |
| Simpson-Kaysen 2019 RP | PTSD | Manualized SUD | FU | 1.14 | 0.06 |  |
| Back 2019 RP | PTSD | Manualized SUD | FU | 1.22 | 0.1 |  |
| McGovern 2011 IAC | PTSD | Manualized SUD | FU | 1.25 | 0.2 |  |
| Ruglass 2017 RP | PTSD | Manualized SUD | FU | 1.29 | 0.08 |  |
| Foa 2013a NAL+SC | PTSD | Manualized SUD | FU | 1.39 | 0.1 |  |
| Foa 2013b PL+SC | PTSD | Manualized SUD | FU | 2.1 | 0.11 |  |
| Hien 2004 Seeking Safety | PTSD | NTF | FU | 0.06 | 0.04 |  |
| Schaefer grant Seeking Safety | PTSD | NTF | FU | 0.31 | 0.01 |  |
| Boden 2011 Seeking Safety | PTSD | NTF | FU | 0.42 | 0.03 |  |
| Possemato 2018 Web CBT + Peer | PTSD | NTF | FU | 0.6 | 0.11 |  |
| Capone 2018 ICBT + TAU | PTSD | NTF | FU | 0.61 | 0.07 |  |
| Myers 2015 SS+ | PTSD | NTF | FU | 0.71 | 0.14 |  |
| Najavits 2018 Seeking Safety | PTSD | NTF | FU | 0.8 | 0.06 |  |
| Brief 2013 VetChange | PTSD | NTF | FU | 0.82 | 0.01 |  |
| Possemato 2018 Web CBT | PTSD | NTF | FU | 0.9 | 0.11 |  |
| Norman 2019 SS | PTSD | NTF | FU | 0.95 | 0.04 |  |
| Zlotnick 2009 SS | PTSD | NTF | FU | 1.06 | 0.05 |  |
| McGovern 2011 ICBT | PTSD | NTF | FU | 1.35 | 0.13 |  |
| Hien 2009 Seeking Safety | PTSD | NTF | FU | 1.43 | 0.01 |  |
| Schaefer grant TAU | PTSD | SUD TAU | FU | 0.44 | 0.01 |  |
| Zlotnick 2009 TAU (SUD) | PTSD | SUD TAU | FU | 0.56 | 0.04 |  |
| van Dam 2013 SUD TAU | PTSD | SUD TAU | FU | 0.61 | 0.08 |  |
| Hamblen 2020 TAU | PTSD | SUD TAU | FU | 0.65 | 0.02 |  |
| Boden 2011 TAU (SUD) | PTSD | SUD TAU | FU | 0.66 | 0.03 |  |
| Capone 2018 TAU | PTSD | SUD TAU | FU | 0.74 | 0.07 |  |
| Hien 2009 Women's Health ED | PTSD | SUD TAU | FU | 1.32 | 0.01 |  |
| Coffey 2016 HLS | PTSD | SUD TAU | FU | 1.37 | 0.06 |  |
| Foa 2013b PE+PL+SC | PTSD | TF v. all | FU | -0.5 | 0.21 |  |
| Haller 2016 CPT | PTSD | TF v. all | FU | -0.41 | 0.08 |  |
| Hamblen 2020 CBT for PTSD | PTSD | TF v. all | FU | -0.27 | 0.05 |  |
| Simpson-Kaysen 2019 CPT | PTSD | TF v. all | FU | -0.12 | 0.08 |  |
| van Dam 2013 SWT | PTSD | TF v. all | FU | -0.07 | 0.16 |  |
| Ruglass 2017 COPE | PTSD | TF v. all | FU | 0.07 | 0.15 |  |
| Sannibale 2013 IT | PTSD | TF v. all | FU | 0.08 | 0.07 |  |
| Back 2019 COPE | PTSD | TF v. all | FU | 0.26 | 0.19 |  |
| Coffey 2016 PE and PE+MET | PTSD | TF v. all | FU | 0.55 | 0.1 |  |
| Foa 2013a PE+NAL+SC | PTSD | TF v. all | FU | 1.08 | 0.28 |  |
| Foa 2013b PE+PL+SC | PTSD | TF v. Manualized SUD | FU | -0.5 | 0.21 |  |
| Haller 2016 CPT | PTSD | TF v. Manualized SUD | FU | -0.41 | 0.08 |  |
| Simpson-Kaysen 2019 CPT | PTSD | TF v. Manualized SUD | FU | -0.12 | 0.08 |  |
| Ruglass 2017 COPE | PTSD | TF v. Manualized SUD | FU | 0.07 | 0.15 |  |
| Sannibale 2013 IT | PTSD | TF v. Manualized SUD | FU | 0.08 | 0.07 |  |
| Back 2019 COPE | PTSD | TF v. Manualized SUD | FU | 0.26 | 0.19 |  |
| Foa 2013a PE+NAL+SC | PTSD | TF v. Manualized SUD | FU | 1.08 | 0.28 |  |
| McGovern 2011 ICBT | PTSD | NTF v. all | FU | -0.58 | 0.27 |  |
| Schaefer grant Seeking Safety | PTSD | NTF v. all | FU | -0.34 | 0.03 |  |
| Boden 2011 Seeking Safety | PTSD | NTF v. all | FU | -0.24 | 0.06 |  |
| Capone 2018 ICBT + TAU | PTSD | NTF v. all | FU | -0.14 | 0.15 |  |
| Hien 2004 Seeking Safety | PTSD | NTF v. all | FU | -0.09 | 0.08 |  |
| Myers 2015 SS+ | PTSD | NTF v. all | FU | -0.04 | 0.32 |  |
| Hien 2009 Seeking Safety | PTSD | NTF v. all | FU | 0.11 | 0.03 |  |
| Zlotnick 2009 SS | PTSD | NTF v. all | FU | 0.19 | 0.07 |  |
| McGovern 2011 ICBT | PTSD | NTF v. Manualized SUD | FU | -0.58 | 0.27 |  |
| Schaefer grant Seeking Safety | PTSD | NTF v. Manualized SUD | FU | -0.34 | 0.03 |  |
| Hien 2004 Seeking Safety | PTSD | NTF v. Manualized SUD | FU | -0.09 | 0.08 |  |
| Myers 2015 SS+ | PTSD | NTF v. Manualized SUD | FU | -0.04 | 0.32 |  |
| Stappenbeck 2015 NC | SUD | No/minimal treatment | post | 0.26 | 0.06 |  |
| Brief 2013 WL | SUD | No/minimal treatment | post | 0.41 | 0.01 |  |
| Simpson-Kaysen 2019 AO | SUD | No/minimal treatment | post | 0.42 | 0.04 |  |
| Ruglass 2017 Monitoring | SUD | No/minimal treatment | post | 0.87 | 0.07 |  |
| Mills 2012 UC in community | SUD | No/minimal treatment | post | 1.11 | 0.03 |  |
| Haller 2016 CPT | SUD | TF | post | -0.26 | 0.02 |  |
| Schacht 2017 PE | SUD | TF | post | 0.22 | 0.04 |  |
| Kehle-Forbes 2018 Integrated MET/PE | SUD | TF | post | 0.25 | 0.01 |  |
| Najavits 2018 Creating Change | SUD | TF | post | 0.31 | 0.04 |  |
| Schacht 2017 PE+CM | SUD | TF | post | 0.32 | 0.04 |  |
| Vujanovic 2018 TIPSS | SUD | TF | post | 0.39 | 0.06 |  |
| Hamblen 2020 CBT for PTSD | SUD | TF | post | 0.51 | 0.03 |  |
| Perez-Dandieu 2014 EMDR | SUD | TF | post | 0.54 | 0.14 |  |
| Kehle-Forbes 2018 Sequential MET/PE | SUD | TF | post | 0.56 | 0.01 |  |
| Ruglass 2017 COPE | SUD | TF | post | 0.6 | 0.06 |  |
| Sannibale 2013 IT | SUD | TF | post | 0.73 | 0.04 |  |
| Simpson-Kaysen 2019 CPT | SUD | TF | post | 0.76 | 0.02 |  |
| Back 2019 COPE | SUD | TF | post | 0.78 | 0.04 |  |
| Norman 2019 PE | SUD | TF | post | 1.21 | 0.04 |  |
| Mills 2012 COPE | SUD | TF | post | 1.33 | 0.03 |  |
| van Dam 2013 SWT | SUD | TF | post | 2.19 | 0.23 |  |
| Foa 2013a PE+NAL+SC | SUD | TF | post | 2.59 | 0.19 |  |
| Foa 2013b PE+PL+SC | SUD | TF | post | 2.95 | 0.22 |  |
| Myers 2015 12SF | SUD | Manualized SUD | post | -0.09 | 0.13 |  |
| Hien 2004 Relapse Prevention | SUD | Manualized SUD | post | 0.07 | 0.04 |  |
| Haller 2016 ICBT | SUD | Manualized SUD | post | 0.07 | 0.02 |  |
| Schaefer grant RP | SUD | Manualized SUD | post | 0.3 | 0.01 |  |
| McGovern 2015 IAC | SUD | Manualized SUD | post | 0.46 | 0.01 |  |
| Vujanovic 2018 CBT | SUD | Manualized SUD | post | 0.62 | 0.04 |  |
| Back 2019 RP | SUD | Manualized SUD | post | 0.62 | 0.08 |  |
| Sannibale 2013 AS | SUD | Manualized SUD | post | 0.83 | 0.04 |  |
| McGovern 2011 IAC | SUD | Manualized SUD | post | 0.89 | 0.08 |  |
| Simpson-Kaysen 2019 RP | SUD | Manualized SUD | post | 1.18 | 0.03 |  |
| Ruglass 2017 RP | SUD | Manualized SUD | post | 1.48 | 0.08 |  |
| Foa 2013b PL+SC | SUD | Manualized SUD | post | 2.62 | 0.17 |  |
| Foa 2013a NAL+SC | SUD | Manualized SUD | post | 3.06 | 0.28 |  |
| Hien 2004 Seeking Safety | SUD | NTF | post | 0.1 | 0.04 |  |
| Possemato 2018 Web CBT | SUD | NTF | post | 0.13 | 0.05 |  |
| Schaefer grant Seeking Safety | SUD | NTF | post | 0.18 | 0.01 |  |
| Possemato 2018 Web CBT + Peer | SUD | NTF | post | 0.25 | 0.07 |  |
| Stappenbeck 2015 EA | SUD | NTF | post | 0.28 | 0.04 |  |
| Stappenbeck 2015 CR | SUD | NTF | post | 0.28 | 0.04 |  |
| Myers 2015 SS+ | SUD | NTF | post | 0.36 | 0.07 |  |
| Capone 2018 ICBT + TAU | SUD | NTF | post | 0.37 | 0.03 |  |
| Hien 2009 Seeking Safety | SUD | NTF | post | 0.38 | 0.01 |  |
| Boden 2011 Seeking Safety | SUD | NTF | post | 0.4 | 0.02 |  |
| Najavits 2018 Seeking Safety | SUD | NTF | post | 0.52 | 0.04 |  |
| McGovern 2015 ICBT | SUD | NTF | post | 0.53 | 0.01 |  |
| McGovern 2011 ICBT | SUD | NTF | post | 0.63 | 0.05 |  |
| Brief 2013 VetChange | SUD | NTF | post | 0.76 | 0.01 |  |
| Norman 2019 SS | SUD | NTF | post | 1.22 | 0.03 |  |
| Perez-Dandieu 2014 TAU (SUD) | SUD | SUD TAU | post | -0.03 | 0.12 |  |
| Schaefer grant TAU | SUD | SUD TAU | post | 0.07 | 0.01 |  |
| Capone 2018 TAU | SUD | SUD TAU | post | 0.25 | 0.03 |  |
| Hamblen 2020 TAU | SUD | SUD TAU | post | 0.25 | 0.02 |  |
| Boden 2011 TAU (SUD) | SUD | SUD TAU | post | 0.26 | 0.02 |  |
| Hien 2009 Women's Health ED | SUD | SUD TAU | post | 0.36 | 0.01 |  |
| McGovern 2015 SC | SUD | SUD TAU | post | 0.48 | 0.01 |  |
| van Dam 2013 SUD TAU | SUD | SUD TAU | post | 1.55 | 0.16 |  |
| Ruglass 2017 COPE | SUD | TF v. all | post | -0.88 | 0.14 |  |
| Foa 2013a PE+NAL+SC | SUD | TF v. all | post | -0.47 | 0.47 |  |
| Haller 2016 CPT | SUD | TF v. all | post | -0.33 | 0.04 |  |
| Simpson-Kaysen 2019 CPT | SUD | TF v. all | post | -0.3 | 0.05 |  |
| Vujanovic 2018 TIPSS | SUD | TF v. all | post | -0.26 | 0.09 |  |
| Sannibale 2013 IT | SUD | TF v. all | post | -0.16 | 0.07 |  |
| Back 2019 COPE | SUD | TF v. all | post | 0.15 | 0.12 |  |
| Hamblen 2020 CBT for PTSD | SUD | TF v. all | post | 0.26 | 0.05 |  |
| Foa 2013b PE+PL+SC | SUD | TF v. all | post | 0.32 | 0.39 |  |
| Perez-Dandieu 2014 EMDR | SUD | TF v. all | post | 0.57 | 0.26 |  |
| van Dam 2013 SWT | SUD | TF v. all | post | 0.58 | 0.25 |  |
| Ruglass 2017 COPE | SUD | TF v. Manualized SUD | post | -0.88 | 0.14 |  |
| Foa 2013a PE+NAL+SC | SUD | TF v. Manualized SUD | post | -0.47 | 0.47 |  |
| Haller 2016 CPT | SUD | TF v. Manualized SUD | post | -0.33 | 0.04 |  |
| Simpson-Kaysen 2019 CPT | SUD | TF v. Manualized SUD | post | -0.3 | 0.05 |  |
| Vujanovic 2018 TIPSS | SUD | TF v. Manualized SUD | post | -0.26 | 0.09 |  |
| Sannibale 2013 IT | SUD | TF v. Manualized SUD | post | -0.16 | 0.07 |  |
| Back 2019 COPE | SUD | TF v. Manualized SUD | post | 0.15 | 0.12 |  |
| Foa 2013b PE+PL+SC | SUD | TF v. Manualized SUD | post | 0.32 | 0.39 |  |
| Schaefer grant Seeking Safety | SUD | NTF v. all | post | -0.12 | 0.02 |  |
| McGovern 2011 ICBT | SUD | NTF v. all | post | -0.04 | 0.17 |  |
| Hien 2009 Seeking Safety | SUD | NTF v. all | post | 0 | 0.02 |  |
| Stappenbeck 2015 CR | SUD | NTF v. all | post | 0.02 | 0.09 |  |
| Hien 2004 Seeking Safety | SUD | NTF v. all | post | 0.03 | 0.08 |  |
| Boden 2011 Seeking Safety | SUD | NTF v. all | post | 0.14 | 0.04 |  |
| Capone 2018 ICBT + TAU | SUD | NTF v. all | post | 0.15 | 0.07 |  |
| Brief 2013 VetChange | SUD | NTF v. all | post | 0.34 | 0.02 |  |
| Myers 2015 SS+ | SUD | NTF v. all | post | 0.46 | 0.2 |  |
| McGovern 2015 ICBT | SUD | NTF v. all | post | 0.47 | 0.06 |  |
| Schaefer grant Seeking Safety | SUD | NTF v. Manualized SUD | post | -0.12 | 0.02 |  |
| McGovern 2011 ICBT | SUD | NTF v. Manualized SUD | post | -0.04 | 0.17 |  |
| Hien 2004 Seeking Safety | SUD | NTF v. Manualized SUD | post | 0.03 | 0.08 |  |
| Myers 2015 SS+ | SUD | NTF v. Manualized SUD | post | 0.46 | 0.2 |  |
| McGovern 2015 ICBT | SUD | NTF v. Manualized SUD | post | 0.47 | 0.06 |  |
| Mills 2012 UC in community | SUD | No/minimal treatment | FU | 1.29 | 0.04 |  |
| Haller 2016 CPT | SUD | TF | FU | -0.48 | 0.03 |  |
| Schacht 2017 PE | SUD | TF | FU | 0.3 | 0.04 |  |
| Hamblen 2020 CBT for PTSD | SUD | TF | FU | 0.34 | 0.03 |  |
| Kehle-Forbes 2018 Integrated MET/PE | SUD | TF | FU | 0.4 | 0.01 |  |
| Back 2019 COPE | SUD | TF | FU | 0.41 | 0.06 |  |
| Najavits 2018 Creating Change | SUD | TF | FU | 0.56 | 0.07 |  |
| Schacht 2017 PE+CM | SUD | TF | FU | 0.58 | 0.05 |  |
| Sannibale 2013 IT | SUD | TF | FU | 0.71 | 0.04 |  |
| Simpson-Kaysen 2019 CPT | SUD | TF | FU | 0.72 | 0.03 |  |
| Kehle-Forbes 2018 Sequential MET/PE | SUD | TF | FU | 0.9 | 0.02 |  |
| Ruglass 2017 COPE | SUD | TF | FU | 0.94 | 0.05 |  |
| Norman 2019 PE | SUD | TF | FU | 1.19 | 0.04 |  |
| van Dam 2013 SWT | SUD | TF | FU | 1.29 | 0.12 |  |
| Mills 2012 COPE | SUD | TF | FU | 1.46 | 0.04 |  |
| Coffey 2016 PE+MET | SUD | TF | FU | 1.93 | 0.07 |  |
| Coffey 2016 PE | SUD | TF | FU | 2.06 | 0.07 |  |
| Foa 2013b PE+PL+SC | SUD | TF | FU | 2.48 | 0.18 |  |
| Foa 2013a PE+NAL+SC | SUD | TF | FU | 2.51 | 0.19 |  |
| Hien 2004 Relapse Prevention | SUD | Manualized SUD | FU | -0.06 | 0.05 |  |
| Haller 2016 ICBT | SUD | Manualized SUD | FU | 0.03 | 0.04 |  |
| Myers 2015 12SF | SUD | Manualized SUD | FU | 0.12 | 0.07 |  |
| Schaefer grant RP | SUD | Manualized SUD | FU | 0.52 | 0.01 |  |
| Back 2019 RP | SUD | Manualized SUD | FU | 0.71 | 0.09 |  |
| McGovern 2011 IAC | SUD | Manualized SUD | FU | 0.89 | 0.09 |  |
| Simpson-Kaysen 2019 RP | SUD | Manualized SUD | FU | 0.93 | 0.04 |  |
| Sannibale 2013 AS | SUD | Manualized SUD | FU | 1.09 | 0.05 |  |
| Ruglass 2017 RP | SUD | Manualized SUD | FU | 1.48 | 0.08 |  |
| Foa 2013b PL+SC | SUD | Manualized SUD | FU | 1.51 | 0.07 |  |
| Foa 2013a NAL+SC | SUD | Manualized SUD | FU | 1.98 | 0.15 |  |
| Myers 2015 SS+ | SUD | NTF | FU | -0.35 | 0.12 |  |
| Capone 2018 ICBT + TAU | SUD | NTF | FU | -0.07 | 0.04 |  |
| Hien 2004 Seeking Safety | SUD | NTF | FU | 0 | 0.04 |  |
| Hien 2009 Seeking Safety | SUD | NTF | FU | 0.12 | 0.01 |  |
| Possemato 2018 Web CBT + Peer | SUD | NTF | FU | 0.22 | 0.07 |  |
| Schaefer grant Seeking Safety | SUD | NTF | FU | 0.25 | 0.01 |  |
| Possemato 2018 Web CBT | SUD | NTF | FU | 0.53 | 0.06 |  |
| Boden 2011 Seeking Safety | SUD | NTF | FU | 0.53 | 0.02 |  |
| Zlotnick 2009 SS | SUD | NTF | FU | 0.57 | 0.04 |  |
| McGovern 2011 ICBT | SUD | NTF | FU | 0.58 | 0.05 |  |
| Najavits 2018 Seeking Safety | SUD | NTF | FU | 0.67 | 0.05 |  |
| Brief 2013 VetChange | SUD | NTF | FU | 1.11 | 0.01 |  |
| Norman 2019 SS | SUD | NTF | FU | 1.19 | 0.04 |  |
| Hien 2009 Women's Health ED | SUD | SUD TAU | FU | 0.04 | 0.01 |  |
| Schaefer grant TAU | SUD | SUD TAU | FU | 0.14 | 0.01 |  |
| Capone 2018 TAU | SUD | SUD TAU | FU | 0.28 | 0.04 |  |
| Boden 2011 TAU (SUD) | SUD | SUD TAU | FU | 0.33 | 0.02 |  |
| Hamblen 2020 TAU | SUD | SUD TAU | FU | 0.4 | 0.02 |  |
| Zlotnick 2009 TAU (SUD) | SUD | SUD TAU | FU | 0.53 | 0.04 |  |
| van Dam 2013 SUD TAU | SUD | SUD TAU | FU | 1.12 | 0.12 |  |
| Coffey 2016 HLS | SUD | SUD TAU | FU | 1.66 | 0.05 |  |
| Ruglass 2017 COPE | SUD | TF v. all | FU | -0.53 | 0.14 |  |
| Haller 2016 CPT | SUD | TF v. all | FU | -0.51 | 0.07 |  |
| Sannibale 2013 IT | SUD | TF v. all | FU | -0.33 | 0.08 |  |
| Back 2019 COPE | SUD | TF v. all | FU | -0.3 | 0.15 |  |
| Simpson-Kaysen 2019 CPT | SUD | TF v. all | FU | -0.12 | 0.06 |  |
| Hamblen 2020 CBT for PTSD | SUD | TF v. all | FU | -0.05 | 0.05 |  |
| van Dam 2013 SWT | SUD | TF v. all | FU | 0.17 | 0.24 |  |
| Coffey 2016 PE and PE+MET | SUD | TF v. all | FU | 0.37 | 0.09 |  |
| Foa 2013a PE+NAL+SC | SUD | TF v. all | FU | 0.53 | 0.34 |  |
| Foa 2013b PE+PL+SC | SUD | TF v. all | FU | 0.96 | 0.25 |  |
| Ruglass 2017 COPE | SUD | TF v. Manualized SUD | FU | -0.53 | 0.14 |  |
| Haller 2016 CPT | SUD | TF v. Manualized SUD | FU | -0.51 | 0.07 |  |
| Sannibale 2013 IT | SUD | TF v. Manualized SUD | FU | -0.33 | 0.08 |  |
| Back 2019 COPE | SUD | TF v. Manualized SUD | FU | -0.3 | 0.15 |  |
| Simpson-Kaysen 2019 CPT | SUD | TF v. Manualized SUD | FU | -0.12 | 0.06 |  |
| Foa 2013a PE+NAL+SC | SUD | TF v. Manualized SUD | FU | 0.53 | 0.34 |  |
| Foa 2013b PE+PL+SC | SUD | TF v. Manualized SUD | FU | 0.96 | 0.25 |  |
| Myers 2015 SS+ | SUD | NTF v. all | FU | -0.47 | 0.18 |  |
| Schaefer grant Seeking Safety | SUD | NTF v. all | FU | -0.27 | 0.02 |  |
| Capone 2018 ICBT + TAU | SUD | NTF v. all | FU | -0.15 | 0.09 |  |
| McGovern 2011 ICBT | SUD | NTF v. all | FU | -0.02 | 0.18 |  |
| Zlotnick 2009 SS | SUD | NTF v. all | FU | 0.04 | 0.07 |  |
| Hien 2004 Seeking Safety | SUD | NTF v. all | FU | 0.06 | 0.08 |  |
| Hien 2009 Seeking Safety | SUD | NTF v. all | FU | 0.07 | 0.02 |  |
| Boden 2011 Seeking Safety | SUD | NTF v. all | FU | 0.21 | 0.04 |  |
| Myers 2015 SS+ | SUD | NTF v. Manualized SUD | FU | -0.47 | 0.18 |  |
| Schaefer grant Seeking Safety | SUD | NTF v. Manualized SUD | FU | -0.27 | 0.02 |  |
| McGovern 2011 ICBT | SUD | NTF v. Manualized SUD | FU | -0.02 | 0.18 |  |
| Hien 2004 Seeking Safety | SUD | NTF v. Manualized SUD | FU | 0.06 | 0.08 |  |

Note: SUD = substance use disorder symptoms; all = all comparators (other than trauma-focused and non-trauma-focused treatment); TF = trauma-focused treatment; NTF = integrated non-trauma-focused treatment; Manualized SUD = CBT manualized SUD treatment; SUD TAU = SUD treatment-as-usual; NAL = Naltrexone; PL = placebo; v. = versus; FU = follow-up.

^a^ Please see Supplemental Materials Table 4 for the full names of each active treatment and control condition.

^1^Studies that were unpublished when the analyses were conducted.

Table A.7. Measures assessing PTSD and substance use disorder symptoms by study

| Study | Outcome | Outcome Type |
| --- | --- | --- |
| Back 2019 | TLFB Percent days drinking/using | SUD |
| Back 2019 | PCL PTSD severity | PTSD |
| Back 2019 | CAPS PTSD severity | PTSD |
| Boden 2011 | ASI Alcohol composite | SUD |
| Boden 2011 | ASI Drug composite | SUD |
| Boden 2011 | IES PTSD severity | PTSD |
| Brief 2013 | QDS Drinks per drinking day | SUD |
| Brief 2013 | QDS Avg. drinks per week | SUD |
| Brief 2013 | QDS Percent days heavy drinking | SUD |
| Brief 2013 | SIP Drinking consequences | SUD |
| Brief 2013 | PCL PTSD severity | SUD |
| Brief 2013 | PTSD Drinking consequences | SUD |
| Capone 2018 | CAPS PTSD severity | PTSD |
| Capone 2018 | TLFB Percent days used alcohol | SUD |
| Capone 2018 | TLFB Percent days used drugs | SUD |
| Capone 2018 | TLFB Percent days abstinent alcohol/drugs | SUD |
| Capone 2018 | ASI Alcohol composite | SUD |
| Capone 2018 | ASI Drug composite | SUD |
| Capone 2018 | toxicology (urine & breathalyzer) positive toxicology screen | SUD |
| Coffey 2016 | IES-R PTSD severity | PTSD |
| Coffey 2016 | TLFB Percent days abstinent; alcohol | SUD |
| Coffey 2016 | TLFB Percent days abstinent; drug | SUD |
| Foa 2013 | TLFB Percent days drinking | SUD |
| Foa 2013 | PSS-I PTSD severity | PTSD |
| Haller 2016 | TLFB Percent days abstinent | SUD |
| Haller 2016 | PCL PTSD severity | PTSD |
| Hamblen 2020 | CAPS PTSD severity | PTSD |
| Hamblen 2020 | ASI Alcohol severity score | SUD |
| Hamblen 2020 | ASI Drug severity score | SUD |
| Hamblen 2020 | PCL PTSD severity | PTSD |
| Hien 2004 | PTSD standardized composite score PTSD severity | PTSD |
| Hien 2004 | SUD standardized composite score SUD severity | SUD |
| Hien 2009 | Substance use inventory days of drug use per week | SUD |
| Hien 2009 | Substance use inventory abstinence rate | SUD |
| Hien 2009 | PTSS PTSD severity | PTSD |
| Hien 2009 | CAPS PTSD severity | PTSD |
| Kehle-Forbes 2018 | PCL PTSD severity | PTSD |
| Kehle-Forbes 2018 | PSS-I PTSD severity | PTSD |
| Kehle-Forbes 2018 | TLFB Percent days heavy drinking/drug use | SUD |
| Kehle-Forbes 2018 | SIP Alcohol problem severity | SUD |
| McGovern 2011 | ASI Alcohol composite | SUD |
| McGovern 2011 | ASI Drug composite | SUD |
| McGovern 2011 | TLFB Days alcohol used | SUD |
| McGovern 2011 | TLFB Days drugs used | SUD |
| McGovern 2011 | Breathalyzer positive BAC | SUD |
| McGovern 2011 | UDS Positive drug screen | SUD |
| McGovern 2011 | CAPS PTSD severity | PTSD |
| McGovern 2011 | CAPS PTSD diagnosis | PTSD |
| McGovern 2015 | ASI Alcohol composite | SUD |
| McGovern 2015 | ASI Drug composite | SUD |
| McGovern 2015 | TLFB Days alcohol used (out of 90) | SUD |
| McGovern 2015 | TLFB Days drugs used | SUD |
| McGovern 2015 | UDS Positive drug screen | SUD |
| McGovern 2015 | CAPS PTSD severity | PTSD |
| Mills 2012 | Opiate Treatment Index No. drug classes used | SUD |
| Mills 2012 | Composite International Diagnostic Interview SUD severity (No. symptoms) | SUD |
| Mills 2012 | CAPS PTSD severity | PTSD |
| Myers 2015 | TLFB Percent days drinking | SUD |
| Myers 2015 | CAPS PTSD severity | PTSD |
| Najavits 2018 | ASI Alcohol severity score | SUD |
| Najavits 2018 | ASI Drug severity score | SUD |
| Najavits 2018 | PCL PTSD severity | PTSD |
| Najavits 2018 | MINI PTSD diagnosis | PTSD |
| Norman 2019 | CAPS PTSD symptom severity | PTSD |
| Norman 2019 | TLFB Percent heavy drinking days | SUD |
| Norman 2019 | TLFB Percent days abstinent | SUD |
| Perez-Dandieu 2014 | ASI SUD severity | SUD |
| Perez-Dandieu 2014 | PCL PTSD severity | PTSD |
| Possemato 2018 | TLFB Percent days heavy drinking | SUD |
| Possemato 2018 | TLFB Percent days drinking | SUD |
| Possemato 2018 | TLFB Percent days drug use | SUD |
| Possemato 2018 | PCL PTSD severity | PTSD |
| Ruglass 2017 | CAPS PTSD severity | PTSD |
| Ruglass 2017 | ASI Days used out of past 30 days | SUD |
| Sannibale 2013 | TLFB Drinks per drinking day | SUD |
| Sannibale 2013 | TLFB Percent days abstinent | SUD |
| Sannibale 2013 | SIP Alcohol consequences | SUD |
| Sannibale 2013 | SCID Alcohol diagnosis | SUD |
| Sannibale 2013 | CAPS PTSD severity | PTSD |
| Sannibale 2013 | CAPS PTSD diagnosis | PTSD |
| Sannibale 2013 | PDS PTSD severity | PTSD |
| Schacht 2017 | UDS Any drug use | SUD |
| Schacht 2017 | CAPS PTSD severity | PTSD |
| Schacht 2017 | UDS Cocaine positive | SUD |
| Schaefer 2019 | ASI-Lite + use item assessing days without use | SUD |
| Schaefer 2019 | ASI-Lite Alcohol severity score | SUD |
| Schaefer 2019 | ASI-Lite Drug severity score | SUD |
| Schaefer 2019 | PSS-I PTSD severity | PTSD |
| Simpson 2021^1^ | CAPS PTSD severity | PTSD |
| Simpson 2021^1^ | CAPS PTSD remission | PTSD |
| Simpson 2021^1^ | Form-90 Drinking days (past 30 days) | SUD |
| Simpson 2021^1^ | Form-90 Heavy drinking days (past 30 days) | SUD |
| Simpson 2021^1^ | Form-90 Low risk drinking | SUD |
| Stappenbeck 2015 | IVR Drinks per day | SUD |
| Stappenbeck 2015 | IVR Average PTSD severity (from PCL) | PTSD |
| van Dam 2013 | TLFB No. abstinent days (past 90 days) | SUD |
| van Dam 2013 | SCID All SUD diagnoses in remission | SUD |
| van Dam 2013 | PDS PTSD severity | PTSD |
| van Dam 2013 | SCID PTSD diagnosis | PTSD |
| Vujanovic 2018^1^ | TLFB Percentage days using primary drug | SUD |
| Vujanovic 2018^1^ | UDS Positive screen for primary drug | SUD |
| Vujanovic 2018^1^ | TLFB Longest sustained abstinence (No. days) | SUD |
| Vujanovic 2018^1^ | CAPS PTSD severity | PTSD |
| Vujanovic 2018^1^ | PCL PTSD severity | PTSD |
| Zlotnick 2009 | ASI Drug composite | SUD |
| Zlotnick 2009 | ASI Alcohol composite | SUD |
| Zlotnick 2009 | CAPS PTSD severity | PTSD |
| Zlotnick 2009 | CAPS PTSD diagnosis | PTSD |
| Zlotnick 2009 | TSC PTSD severity | PTSD |

Note: ASI = Addiction Severity Index; CAPS = Clinician Administered PTSD Scale; BAC = blood alcohol content; IES = Impact of Event Scale; IES-R = Impact of Event Scale, Revised; IVR = Interactive Voice Response (daily data collection); MINI = MINI International Neuropsychiatry Interview; PCL = PTSD Checklist; PDS = Posttraumatic Diagnostic Scale; PSS-I = PTSD Symptom Scale – Interview; PTSS = Posttraumatic Stress Syndrome Scale; QDS = Quick Drinking Scale; SCID = Structured Interview for DSM Disorders; SIP = Short Inventory of Problems (SUD); TLFB = Timeline Follow-back; TSC = Trauma Symptom Checklist; UDS = urine drug screen.

^1^Studies that were unpublished when the analyses were conducted.

Table A.8. Results of moderator tests pertaining to attrition, group/individual, DUD rates, and recruitment site

| Treatment / Comparison | Domain | Time | B_attr_ | p_attr_ | B_group_ | p_group_ | B_dud_ | p_dud_ | B_sudsite_ | p_sudsite_ |
| --- | --- | --- | --- | --- | --- | --- | --- | --- | --- | --- |
| **Within Condition Type** |  |  |  |  |  |  |  |  |  |  |
| Trauma-focused | PTSD | post | 0.78 | .236 | NA | NA | 0.00 | .736 | 0.38 | .134 |
| Trauma-focused | SUD | post | **2.52** | **<.001** | NA | NA | 0.00 | .824 | 0.59 | .103 |
| Trauma-focused | PTSD | FU | 1.11 | .180 | NA | NA | 0.00 | .592 | 0.33 | .268 |
| Trauma-focused | SUD | FU | 0.81 | .434 | NA | NA | 0.00 | .847 | **0.68** | **.045** |
| Non-trauma-focused | PTSD | post | 0.65 | .336 | 0.06 | .807 | -0.01 | .627 | **0.48** | **.016** |
| Non-trauma-focused | SUD | post | 0.38 | .487 | -0.1 | .657 | 0.00 | .378 | -0.08 | .649 |
| Non-trauma-focused | PTSD | FU | 0.31 | .677 | 0.23 | .406 | 0.00 | .759 | 0.3 | .269 |
| Non-trauma-focused | SUD | FU | 0.28 | .703 | -0.14 | .636 | 0.00 | .908 | -0.24 | .440 |
| Manualized SUD | PTSD | post | 0.87 | .181 | NA | NA | -0.01 | .406 | 0.5 | .059 |
| Manualized SUD | SUD | post | **2.39** | **.024** | NA | NA | -0.01 | .506 | **1.01** | **.044** |
| Manualized SUD | PTSD | FU | **1.80** | **.024** | NA | NA | -0.01 | .518 | **0.77** | **.017** |
| Manualized SUD | SUD | FU | 1.14 | .342 | NA | NA | -0.01 | .36 | **0.84** | **.041** |
| SUD TAU | PTSD | post | 0.42 | .665 | 0.03 | .937 | 0.00 | .849 | NA | NA |
| SUD TAU | SUD | post | -0.12 | .845 | -0.3 | .504 | 0.00 | .301 | NA | NA |
| SUD TAU | PTSD | FU | 0.31 | .792 | -0.14 | .747 | 0.01 | .501 | NA | NA |
| SUD TAU | SUD | FU | -1.9 | .215 | **-1.19** | **<.001** | -0.01 | .450 | NA | NA |
| No/minimal treatment | PTSD | post | -0.87 | .127 | NA | NA | 0.00 | .812 | NA | NA |
| No/minimal treatment | SUD | post | 0.31 | .783 | NA | NA | **0.02** | **.007** | NA | NA |
| No/minimal treatment | PTSD | FU | NA | NA | NA | NA | NA | NA | NA | NA |
| No/minimal treatment | SUD | FU | NA | NA | NA | NA | NA | NA | NA | NA |
| **Between Condition Types** |  |  |  |  |  |  |  |  |  |  |
| Trauma-focused v. all | PTSD | post | -0.22 | .712 | NA | NA | 0.00 | .813 | 0.37 | .096 |
| Trauma-focused v. all | SUD | post | 0.37 | .503 | NA | NA | 0.00 | .425 | **0.58** | **.006** |
| Trauma-focused v. all | PTSD | FU | -0.18 | .808 | NA | NA | 0.00 | .268 | 0.13 | .623 |
| Trauma-focused v. all | SUD | FU | 0.23 | .774 | NA | NA | 0.00 | .598 | **0.55** | **.006** |
| Trauma-focused v. Manualized SUD | PTSD | post | 0.41 | .488 | NA | NA | -0.01 | .399 | 0.20 | .555 |
| Trauma-focused v. Manualized SUD | SUD | post | 0.28 | .650 | NA | NA | -0.01 | .203 | 0.25 | .602 |
| Trauma-focused v. Manualized SUD | PTSD | FU | 0.66 | .515 | NA | NA | -0.01 | .392 | 0.24 | .533 |
| Trauma-focused v. Manualized SUD | SUD | FU | 1.46 | .13 | NA | NA | -0.01 | .496 | **1.11** | **.006** |
| Non-trauma-focused v. all | PTSD | post | 0.31 | .524 | -0.23 | .148 | -0.01 | .159 | -0.16 | .307 |
| Non-trauma-focused v. all | SUD | post | 0.13 | .830 | -0.24 | .081 | 0.00 | .284 | -0.17 | .221 |
| Non-trauma-focused v. all | PTSD | FU | -0.42 | .598 | 0.22 | .411 | 0.01 | .292 | 0.07 | .808 |
| Non-trauma-focused v. all | SUD | FU | -0.57 | .400 | 0.18 | .424 | 0.00 | .524 | 0.18 | .493 |
| Non-trauma-focused v. Manualized SUD | PTSD | post | 1.12 | .532 | NA | NA | NA | NA | 0.36 | .494 |
| Non-trauma-focused v. Manualized SUD | SUD | post | -0.55 | .686 | NA | NA | -0.01 | .676 | 0.18 | .569 |
| Non-trauma-focused v. Manualized SUD | PTSD | FU | 0.28 | .824 | NA | NA | NA | NA | -0.51 | .381 |
| Non-trauma-focused v. Manualized SUD | SUD | FU | -0.14 | .883 | NA | NA | NA | NA | 0.09 | .861 |

Note: Bolded text indicates *p* < .05; B_attr_ = meta-regression coefficient predicting effect sizes from attrition; p_attr_ = p-value for B_attr_; B_dud_ = meta-regression coefficient predicting effect sizes from percentage of sample reporting drug use; p_dud_ = p-value for B_dud_; B_sudsite_ = meta-regression coefficient predicting effect sizes from recruitment setting (i.e., SUD treatment clinics vs. community-based recruitment); p_sudsite_ = p-value for B_sudsite_; NA = not available due to insufficient studies with data on predictor.

Table A.9. Integrated vs. non-integrated treatment as a moderator for trauma-focused treatments

| Comparison | Model | Domain | Time | B_integ_ | p_integ_ | B_adj_ | p_adj_ |  |
| --- | --- | --- | --- | --- | --- | --- | --- | --- |
| Trauma | Within-group | PTSD | post | **-0.54** | **.022** | -0.49 | .353 |  |
| Trauma | Within-group | SUD | post | -0.65 | .082 | -0.17 | .825 |  |
| Trauma | Within-group | PTSD | FU | -0.46 | .109 | -0.21 | .742 |  |
| Trauma | Within-group | SUD | FU | **-0.74** | **.032** | -0.05 | .949 |  |
| Trauma v. all | Between-group | PTSD | post | **-0.46** | **.019** | -0.26 | .461 |  |
| Trauma v. all | Between-group | SUD | post | -0.26 | .277 | 0.02 | .925 |  |
| Trauma v. all | Between-group | PTSD | FU | -0.18 | .510 | 0.08 | .847 |  |
| Trauma v. all | Between-group | SUD | FU | **-0.63** | **.005** | -0.30 | .302 |  |
| Trauma v. Manual SUD | Between-group | PTSD | post | -0.27 | .241 | -0.26 | .462 |  |
| Trauma v. Manual SUD | Between-group | SUD | post | -0.02 | .916 | 0.02 | .925 |  |
| Trauma v. Manual SUD | Between-group | PTSD | FU | -0.07 | .836 | 0.09 | .854 |  |
| Trauma v. Manual SUD | Between-group | SUD | FU | **-0.57** | **.028** | -0.30 | .302 |  |

Note: Bolded text indicates *p* < .05; B_integ_ = meta-regression coefficient predicting effect sizes from use of integrated trauma-focused treatment; p_integ_ = *p*-value for B_integ_; B_adj_ = meta-regression coefficient for use of integrated trauma-focused treatment when controlling for recruitment setting (i.e., SUD treatment clinics vs. community-based recruitment); p_adj_ = *p*-value for B_adj_; Trauma = trauma-focused treatment; v. = versus; Manual SUD = manualized SUD treatment; FU = follow-up.

Table A.10. Models results with studies including subthreshold SUD excluded

| Treatment | Domain | Time | k | ES | I^2^ |
| --- | --- | --- | --- | --- | --- |
| Trauma | PTSD | post | 17 | 1.11 [0.86, 1.35] | 85.37 [73.66, 93.62] |
| Trauma | SUD | post | 16 | 0.82 [0.47, 1.16] | 93.55 [88.77, 97.77] |
| Trauma | PTSD | FU | 15 | 1.10 [0.81, 1.39] | 87.49 [77.34, 94.85] |
| Trauma | SUD | FU | 15 | 0.97 [0.62, 1.33] | 92.98 [87.45, 97.18] |
| Non-trauma | PTSD | post | 13 | 0.64 [0.43, 0.86] | 79.18 [60.66, 93.09] |
| Non-trauma | SUD | post | 12 | 0.41 [0.26, 0.56] | 66.03 [30.57, 87.20] |
| Non-trauma | PTSD | FU | 11 | 0.75 [0.50, 1.01] | 77.76 [52.88, 91.14] |
| Non-trauma | SUD | FU | 11 | 0.37 [0.15, 0.59] | 79.08 [55.80, 93.74] |
| Manual SUD | PTSD | post | 13 | 0.85 [0.58, 1.11] | 79.26 [56.96, 91.99] |
| Manual SUD | SUD | post | 13 | 0.87 [0.41, 1.33] | 94.70 [89.48, 98.46] |
| Manual SUD | PTSD | FU | 11 | 1.01 [0.70, 1.31] | 75.30 [45.91, 92.13] |
| Manual SUD | SUD | FU | 11 | 0.80 [0.43, 1.18] | 87.61 [73.58, 96.32] |
| SUD TAU | PTSD | post | 10 | 0.58 [0.30, 0.86] | 85.71 [68.45, 96.28] |
| SUD TAU | SUD | post | 8 | 0.30 [0.16, 0.45] | 54.15 [26.95, 97.93] |
| SUD TAU | PTSD | FU | 8 | 0.79 [0.54, 1.05] | 77.46 [47.09, 94.21] |
| SUD TAU | SUD | FU | 8 | 0.52 [0.16, 0.88] | 91.73 [79.59, 98.25] |
| No tx | PTSD | post | 4 | 0.56 [0.29, 0.83] | 37.34 [0.00, 94.70] |
| No tx | SUD | post | 4 | 0.67 [0.28, 1.07] | 72.88 [18.17, 97.95] |
| No tx | PTSD | FU | 1 | 1.04 [0.65, 1.43] | NA |
| No tx | SUD | FU | 1 | 1.29 [0.92, 1.66] | NA |
| Trauma v. all | PTSD | post | 12 | 0.29 [0.07, 0.52] | 32.95 [0.00, 91.21] |
| Trauma v. all | SUD | post | 11 | -0.11 [-0.34, 0.11] | 25.34 [0.00, 82.18] |
| Trauma v. all | PTSD | FU | 10 | -0.01 [-0.24, 0.22] | 20.95 [0.00, 83.54] |
| Trauma v. all | SUD | FU | 10 | -0.09 [-0.32, 0.14] | 21.72 [0.00, 85.28] |
| Trauma v. Manual SUD | PTSD | post | 8 | 0.15 [-0.06, 0.37] | 4.39 [0.00, 88.82] |
| Trauma v. Manual SUD | SUD | post | 8 | -0.27 [-0.48, -0.06] | 0.00 [0.00, 78.83] |
| Trauma v. Manual SUD | PTSD | FU | 7 | -0.03 [-0.29, 0.23] | 3.06 [0.00, 90.04] |
| Trauma v. Manual SUD | SUD | FU | 7 | -0.21 [-0.47, 0.04] | 6.06 [0.00, 92.27] |
| Non-trauma v. all | PTSD | post | 9 | 0.04 [-0.12, 0.20] | 0.00 [0.00, 35.12] |
| Non-trauma v. all | SUD | post | 9 | 0.05 [-0.09, 0.19] | 0.00 [0.00, 62.51] |
| Non-trauma v. all | PTSD | FU | 8 | -0.10 [-0.30, 0.09] | 12.72 [0.00, 65.79] |
| Non-trauma v. all | SUD | FU | 8 | -0.04 [-0.21, 0.13] | 16.09 [0.00, 68.85] |
| Non-trauma v. Manual SUD | PTSD | post | 4 | 0.01 [-0.25, 0.28] | 0.00 [0.00, 88.45] |
| Non-trauma v. Manual SUD | SUD | post | 5 | 0.10 [-0.18, 0.39] | 33.30 [0.00, 88.80] |
| Non-trauma v. Manual SUD | PTSD | FU | 4 | -0.28 [-0.56, -0.00] | 0.00 [0.00, 85.46] |
| Non-trauma v. Manual SUD | SUD | FU | 4 | -0.21 [-0.44, 0.02] | 0.00 [0.00, 88.80] |

Note: No changes in statistical significance were observed relative to primary analyses. k = number of comparisons contributing to effect size estimate; ES = effect size in Hedges’ *g* units; I^2^ = heterogeneity; SUD = substance use disorder symptoms; Non-trauma = integrated non-trauma-focused treatment; Trauma = trauma-focused treatment; v. = versus; Manual SUD = manualized SUD treatment; FU = follow-up.

Table A.11. Results within- and between-group models with outliers excluded

| Treatment / Comparison | Domain | Time | k | ES | k_out_ | ES_out_ |
| --- | --- | --- | --- | --- | --- | --- |
| Trauma-focused | PTSD | post | 17 | 1.11 [0.86, 1.35] | 4 | 1.08 [0.88, 1.27] |
| Trauma-focused | SUD | post | 16 | 0.82 [0.47, 1.16] | 5 | 0.64 [0.46, 0.83] |
| Trauma-focused | PTSD | FU | 15 | 1.10 [0.81, 1.39] | 4 | 1.20 [0.93, 1.47] |
| Trauma-focused | SUD | FU | 15 | 0.97 [0.62, 1.33] | 6 | 0.77 [0.56, 0.98] |
| Non-trauma-focused | PTSD | post | 14 | 0.65 [0.45, 0.84] | 3 | 0.48 [0.35, 0.62] |
| Non-trauma-focused | SUD | post | 13 | 0.44 [0.29, 0.59] | 2 | 0.35 [0.25, 0.44] |
| Non-trauma-focused | PTSD | FU | 12 | 0.76 [0.53, 0.99] | 2 | 0.73 [0.54, 0.91] |
| Non-trauma-focused | SUD | FU | 12 | 0.43 [0.20, 0.67] | 2 | 0.29 [0.13, 0.46] |
| Manualized SUD | PTSD | post | 13 | 0.85 [0.58, 1.11] | 3 | 1.07 [0.92, 1.22] |
| Manualized SUD | SUD | post | 13 | 0.87 [0.41, 1.33] | 3 | 0.63 [0.36, 0.91] |
| Manualized SUD | PTSD | FU | 11 | 1.01 [0.70, 1.31] | 2 | 0.96 [0.75, 1.17] |
| Manualized SUD | SUD | FU | 11 | 0.80 [0.43, 1.18] | 3 | 0.89 [0.57, 1.21] |
| SUD TAU | PTSD | post | 10 | 0.58 [0.30, 0.86] | 2 | 0.42 [0.21, 0.64] |
| SUD TAU | SUD | post | 8 | 0.30 [0.16, 0.45] | 1 | 0.26 [0.13, 0.39] |
| SUD TAU | PTSD | FU | 8 | 0.79 [0.54, 1.05] | 1 | 0.68 [0.47, 0.88] |
| SUD TAU | SUD | FU | 8 | 0.52 [0.16, 0.88] | 1 | 0.30 [0.13, 0.48] |
| No/minimal treatment | PTSD | post | 5 | 0.49 [0.27, 0.71] | 0 | 0.49 [0.27, 0.71] |
| No/minimal treatment | SUD | post | 5 | 0.61 [0.29, 0.92] | 0 | 0.61 [0.29, 0.92] |
| No/minimal treatment | PTSD | FU | 1 | 1.04 [0.65, 1.43] | 0 | 1.04 [0.65, 1.43] |
| No/minimal treatment | SUD | FU | 1 | 1.29 [0.92, 1.66] | 0 | 1.29 [0.92, 1.66] |
| Trauma-focused v. all | PTSD | post | 12 | 0.29 [0.07, 0.52] | 1 | 0.23 [0.04, 0.43] |
| Trauma-focused v. all | SUD | post | 11 | -0.11 [-0.34, 0.11] | 0 | -0.11 [-0.34, 0.11] |
| Trauma-focused v. all | PTSD | FU | 10 | -0.01 [-0.24, 0.22] | 0 | -0.01 [-0.24, 0.22] |
| Trauma-focused v. all | SUD | FU | 10 | -0.09 [-0.32, 0.14] | 0 | -0.09 [-0.32, 0.14] |
| Trauma-focused v. Manualized SUD | PTSD | post | 8 | 0.15 [-0.06, 0.37] | 0 | 0.15 [-0.06, 0.37] |
| Trauma-focused v. Manualized SUD | SUD | post | 8 | -0.27 [-0.48, -0.06] | 0 | -0.27 [-0.48, -0.06] |
| Trauma-focused v. Manualized SUD | PTSD | FU | 7 | -0.03 [-0.29, 0.23] | 0 | -0.03 [-0.29, 0.23] |
| Trauma-focused v. Manualized SUD | SUD | FU | 7 | -0.21 [-0.47, 0.04] | 0 | -0.21 [-0.47, 0.04] |
| Non-trauma-focused v. all | PTSD | post | 10 | 0.11 [-0.03, 0.26] | 0 | 0.11 [-0.03, 0.26] |
| Non-trauma-focused v. all | SUD | post | 10 | 0.12 [-0.03, 0.28] | 0 | 0.12 [-0.03, 0.28] |
| Non-trauma-focused v. all | PTSD | FU | 8 | -0.10 [-0.30, 0.09] | 0 | -0.10 [-0.30, 0.09] |
| Non-trauma-focused v. all | SUD | FU | 8 | -0.04 [-0.21, 0.13] | 0 | -0.04 [-0.21, 0.13] |
| Non-trauma-focused v. Manualized SUD | PTSD | post | 4 | 0.01 [-0.25, 0.28] | 0 | 0.01 [-0.25, 0.28] |
| Non-trauma-focused v. Manualized SUD | SUD | post | 5 | 0.10 [-0.18, 0.39] | 0 | 0.10 [-0.18, 0.39] |
| Non-trauma-focused v. Manualized SUD | PTSD | FU | 4 | -0.28 [-0.56, 0.00] | 0 | -0.28 [-0.56, 0.00] |
| Non-trauma-focused v. Manualized SUD | SUD | FU | 4 | -0.21 [-0.44, 0.02] | 0 | -0.21 [-0.44, 0.02] |

Note: k = number of treatment or control arms (for within-group models) or comparisons (for between-group models) contributing to effect size estimate; ES = effect size in Hedges’ *g* units; k_out_ = number of outliers detected; ES_out_ = effect size with outliers removed; FU = follow-up; NA = not available due to insufficient studies with data on predictor.

Figure A.1. Cochrane risk of bias ratings aggregated across studies. Allocat Concealment = allocation concealment; Blind Outcome = blinding of outcome assessor; low = low risk of bias; unclear = unclear risk of bias; high = high risk of bias.

Figure A.2. Funnel plot regarding publication bias in treatment retention; non-trauma-focused treatments vs. all comparators

Figure A.3. Funnel plot regarding publication bias in treatment retention; non-trauma-focused treatments vs. manualized SUD treatments
